# Resveratrol Alleviates Arsenic-Induced Liver Fibrosis in Rats by Correcting SIRT1-Mediated Disorder of Hepatic Bile Acid Metabolism

**DOI:** 10.3390/ijms27115123

**Published:** 2026-06-05

**Authors:** Qi Wang, Hualin Chen, Qiming Ran, Fang Hu, Qiwen Fu, Lu Ma

**Affiliations:** 1 The Key Laboratory of Environmental Pollution Monitoring and Disease Control, Ministry of Education, Department of Toxicology, School of Public Health, Guizhou Medical University, Guiyang 561113, China; wqgzykd@163.com (Q.W.); chenhualin528@126.com (H.C.); ranqiming330@163.com (Q.R.); hufang0519@163.com (F.H.); 15885782862@163.com (Q.F.); 2 Collaborative Innovation Center for Prevention and Control of Endemic and Ethnic Regional Diseases Co-Constructed by the Province and Ministry, Guizhou Medical University, Guiyang 561113, China

**Keywords:** arsenic, SIRT1, C/EBPα, bile acid metabolism, liver fibrosis, resveratrol

## Abstract

Liver fibrosis is a reversible phase of arsenic-induced chronic liver injury, with bile acid metabolic alterations closely associated with this pathological process. SIRT1 is a key metabolic regulator and promising therapeutic candidate, while its role in bile acid metabolism during arsenic-induced liver fibrosis remains poorly defined. This study established time-dependent rat models of arsenic-induced liver fibrosis with resveratrol intervention to investigate the potential association between SIRT1, bile acid metabolic disturbance, and liver fibrosis, as well as resveratrol’s hepatoprotective effects. Arsenic exposure induces progressive accumulation of hydrophobic bile acids in the liver, inflammation, hepatic stellate cell activation, and fibrosis, accompanied by suppressed expression of bile acid phase II detoxification genes (*Baat*, *Ugt1a1*, *Sult2a1*) and the bile acid efflux transporter gene *Abcb11* (BSEP). Arsenic exposure reduces SIRT1 expression and increases C/EBPα acetylation, which may relate to impaired transcription of the aforementioned bile acid metabolic genes. Resveratrol may restore SIRT1 expression, normalize C/EBPα acetylation and bile acid homeostasis, and reduce hepatic arsenic accumulation, thereby alleviating liver fibrosis. Collectively, the SIRT1/C/EBPα axis and bile acid metabolism may be linked to the progression of arsenic-induced liver fibrosis. Resveratrol may exert protective effects via multiple mechanisms, including regulating these molecular targets and reducing hepatic arsenic accumulation.

## 1. Introduction

Arsenic is a naturally occurring metalloid in the Earth’s crust, with environmental arsenic contamination stemming from natural geological release, industrial wastewater discharge, coal-burning arsenic emission, and the application of arsenic-containing pesticides in agriculture. Drinking water and food are the primary routes of chronic arsenic exposure to humans [1,2,3]. Currently, more than 200 million people worldwide are at risk of drinking water arsenic contamination, rendering environmental arsenic contamination a major global public health concern [4,5,6]. The liver is the major target organ of arsenic. Epidemiological studies have confirmed that the incidence of abnormal liver function is significantly elevated in arsenic-contaminated areas, and there is a clear positive correlation between arsenic exposure dosage and the risk of liver fibrosis, cirrhosis, and hepatocellular carcinoma [7,8,9,10,11]. Moreover, cirrhosis, ascites, and hepatocellular carcinoma are the main causes of death in patients with arsenic poisoning [12]. Despite the well-established hepatotoxicity of arsenic, effective interventions remain lacking.

Notably, clinical studies from Bangladesh and China have consistently identified liver fibrosis as a typical morphological lesion in chronic arsenic poisoning [8,11,13]. As a critical reversible stage in the progression of liver injury to cirrhosis and hepatocellular carcinoma, preventing or reversing liver fibrosis is a core strategy to block the irreversible progression of chronic liver disease [14,15]. Therefore, exploring the molecular mechanisms of arsenic-induced liver fibrosis and developing targeted interventions are crucial for mitigating the adverse outcomes of environmental arsenic-induced hepatotoxicity.

Liver fibrosis is a reparative response of the liver to chronic injury, characterized by hepatic stellate cell (HSC) activation that leads to increased secretion and excessive deposition of extracellular matrix [16]. Accumulating evidence indicates that hepatic bile acid metabolic dysregulation is closely associated with liver fibrosis. This metabolic disorder can promote HSC activation by inducing intrahepatic and peribiliary inflammatory responses, as well as regulating the pathways of nuclear receptors such as farnesoid X receptor (FXR) and pregnane X receptor (PXR), thus triggering extracellular matrix deposition and facilitating the progression of liver fibrosis [17,18]. In addition, restoring the balance of hepatic bile acid metabolism has been confirmed to effectively inhibit HSC activation, reduce extracellular matrix deposition, and delay or even reverse liver fibrosis, suggesting its potential as an early intervention approach for liver fibrosis [18,19]. However, whether and how arsenic disrupts bile acid metabolism and the association between such changes and liver fibrosis remain poorly understood. This unresolved issue hinders the exploration of novel mechanisms and early therapeutic approaches for arsenic-induced liver fibrosis based on bile acid metabolism.

Silent mating type information regulation 2 homolog 1 (SIRT1), a nicotinamide adenine dinucleotide (NAD^+^)-dependent histone deacetylase, exerts broad regulatory effects on essential physiological processes by mediating the deacetylation of downstream target proteins and is closely implicated in the regulation of hepatic bile acid metabolism [20,21,22]. Clinical studies have revealed that SIRT1 expression levels are significantly reduced in patients with cholestasis [23]. Animal experiments have also shown that inhibiting or knocking out SIRT1 can induce bile acid metabolic disorders. Conversely, activating SIRT1 can alleviate these disorders, restore bile acid homeostasis, and thereby mitigate cholestasis and liver toxicity [24,25,26]. These findings indicate that SIRT1 plays a pivotal role in maintaining hepatic bile acid metabolic homeostasis. However, the linkage between SIRT1 and arsenic-triggered bile acid dysregulation has not been fully elucidated. It also remains to be clarified whether this interaction correlates with the development of arsenic-induced liver fibrosis. Resolving these questions may facilitate the discovery of potential therapeutic targets for this disease.

Resveratrol (RSV), a plant-derived polyphenol and natural agonist of SIRT1, is widely distributed in grapes, nuts, and herbs, with potent anti-inflammatory, antioxidant, and disease-preventive activities [27,28]. Previous studies have shown that RSV ameliorates liver fibrosis by inhibiting hepatocyte apoptosis [29], oxidative stress [30], inflammatory responses [31], and HSC activation [32], yet its role in arsenic-induced liver fibrosis remains unclear. Although RSV is widely acknowledged as a SIRT1 activator, limited studies have explored the potential association between its antifibrotic properties and SIRT1-modulated hepatic bile acid metabolism, which is closely correlated with the early pathological progression of liver fibrosis. Given the promising hepatoprotective potential and favorable safety profile of RSV, we established time-dependent rat models of arsenic-induced liver fibrosis with RSV intervention. Targeted bile acid metabolomics was performed to characterize the key perturbed bile acid metabolic pathways involved in arsenic-induced liver fibrosis, and to clarify the relationship of SIRT1 with these metabolic alterations. On this basis, this study further explored the protective effects and underlying mechanisms of RSV against arsenic-induced hepatic bile acid dysregulation and subsequent liver fibrosis in rats.

## 2. Results

### 2.1. Hepatic Bile Acid Accumulation and Progressive Liver Fibrosis in Rats Following Long-Term Arsenic Exposure

**Figure 1:**
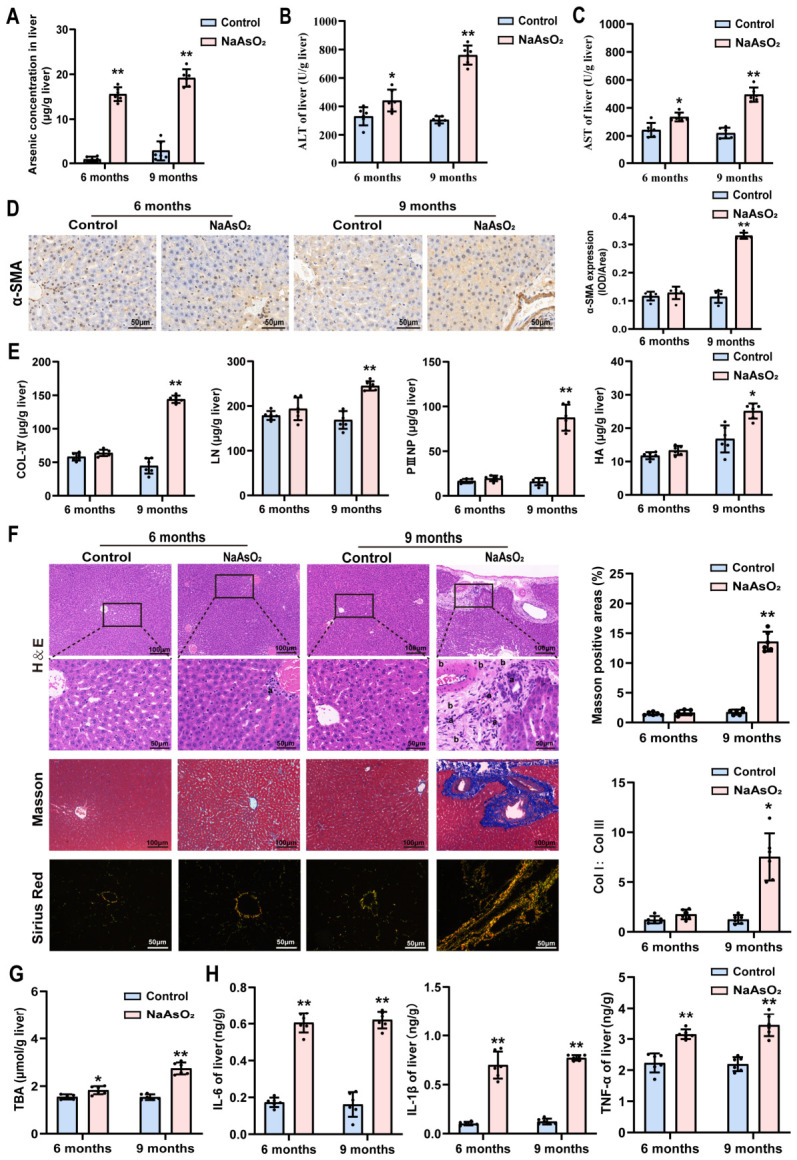
Long-term arsenic exposure induces hepatic bile acid accumulation and triggers liver fibrosis in rats. (**A**) Arsenic concentration in liver. (**B**–**E**) ALT, AST, α-SMA, COL-IV, LN, PIIINP, and HA levels in liver. (**F**) H&E staining, Masson trichrome staining and Sirius Red staining; a: inflammatory infiltration; b: collagen fibers. (**G**,**H**) TBA, IL-6, IL-1β, and TNF-α levels in liver. Data were obtained from rats with 6- and 9-month arsenic exposure and their time-matched controls. All data were shown as mean ± SD (n = 6). Independent-samples *t*-test was used for all predefined pairwise comparisons. * *p* < 0.05, ** *p* < 0.001 vs. time-matched control.

To clarify the association between hepatic bile acid accumulation and arsenic-induced liver fibrosis, a rat model of arsenic exposure with different durations was established. The results showed that 6-month and 9-month arsenic exposure had no significant effect on the body weight or liver coefficient of rats (Figure S1), but significantly increased hepatic arsenic concentration and the activities of liver function indicators alanine aminotransferase (ALT) and aspartate aminotransferase (AST), with a more obvious elevation in the 9-month exposure group (Figure 1A–C). HSC activation and collagen deposition are characteristic hallmarks of liver fibrosis. Compared with the control group, the levels of HSC activation marker α-smooth muscle actin (α-SMA) and collagen-related markers collagen IV (COL-IV), laminin (LN), procollagen III N-terminal propeptide (PIIINP), and hyaluronic acid (HA) in liver tissue showed no significant change in the 6-month arsenic exposure group, while all the aforementioned indicators were significantly upregulated in the 9-month exposure group (Figure 1D,E). Pathological staining results were consistent with this finding. Only inflammatory infiltration was observed in the liver of the 6-month arsenic exposure group without fibrosis, whereas the 9-month exposure group exhibited obvious fibrosis in the vascular area of liver tissue. Masson and Sirius Red staining further confirmed that the collagen fiber deposition area and the ratio of type I to type III collagen in the 9-month arsenic exposure group were significantly increased compared with the control group, with no significant difference in the 6-month exposure group (Figure 1F). These results demonstrate that 9-month arsenic exposure can induce liver fibrosis in rats.

Given that hepatic bile acid accumulation induces HSC activation and excessive collagen secretion by triggering inflammation, the levels of TBA and inflammatory factors in liver tissue were further detected. The results showed that the levels of total bile acid (TBA), interleukin 6 (IL-6), interleukin-1β (IL-1β), and tumor necrosis factor-α (TNF-α) in the 6-month and 9-month arsenic exposure groups were significantly increased compared with the control group (Figure 1G,H). Collectively, these results suggest arsenic exposure for 6 months is sufficient to induce hepatic bile acid accumulation and elevated release of inflammatory factors. As arsenic exposure continued for 9 months, bile acid accumulation became more severe, which was accompanied by persistent release of inflammatory factors, HSC activation and progressive liver fibrosis. Accordingly, we initiated RSV intervention at the 6-month time point of arsenic exposure, to explore whether resveratrol could ameliorate arsenic-induced liver fibrosis and whether such effects were associated with reduced hepatic bile acid accumulation.

### 2.2. Resveratrol Alleviates Arsenic-Induced Hepatic Bile Acid Accumulation and Subsequent Liver Fibrosis

**Figure 2:**
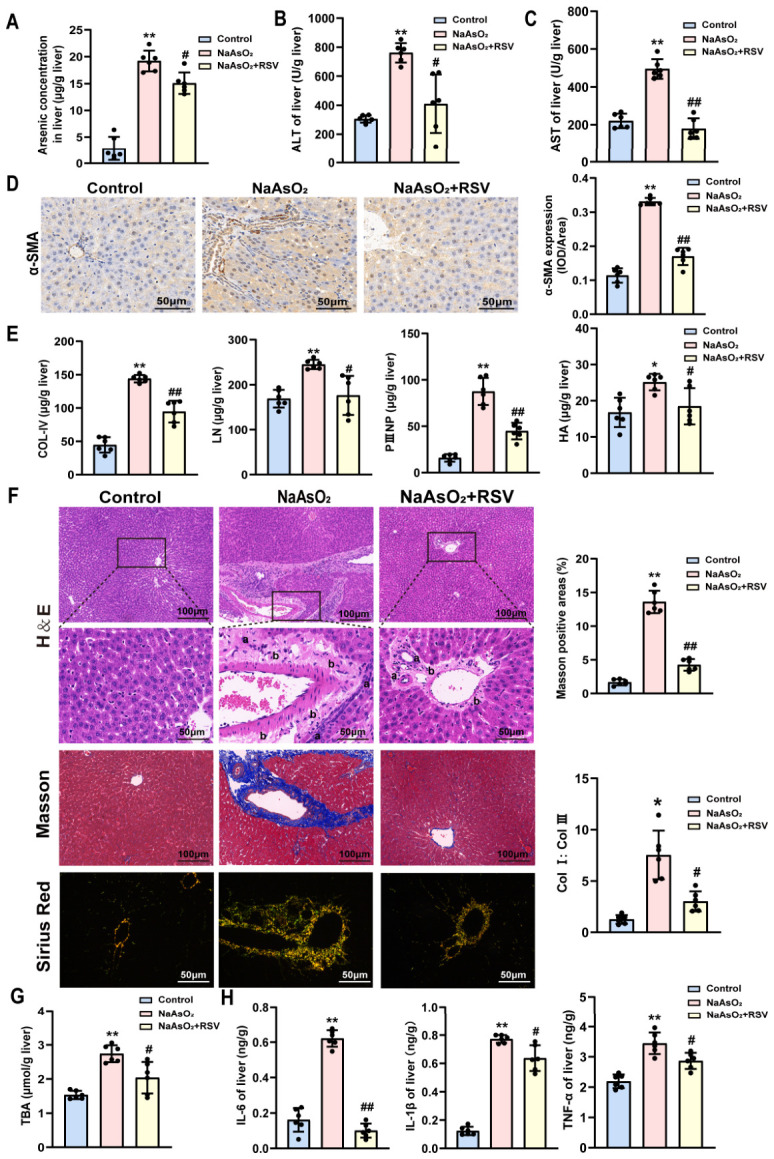
Resveratrol alleviates arsenic-induced liver fibrosis by reducing hepatic bile acid accumulation and subsequent inflammatory response. (**A**) Arsenic concentration in liver. (**B**–**E**) ALT, AST, α-SMA, COL-IV, LN, PIIINP, and HA levels in liver. (**F**) H&E staining, Masson trichrome staining, and Sirius Red staining; a: inflammatory infiltration; b: collagen fibers. (**G**,**H**) TBA, IL-6, IL-1β, and TNF-α levels in liver. Data were obtained from rats exposed to arsenic for 9 months, rats continuously exposed to arsenic and co-treated with resveratrol from month 6 to 9, and their time-matched controls. All data were shown as mean ± SD (n = 6). Independent-samples *t*-test was used for all predefined pairwise comparisons. * *p* < 0.05, ** *p* < 0.001 vs. Control; ^#^ *p* < 0.05, ^##^ *p* < 0.001 vs. NaAsO_2_.

We first evaluated the protective effect of RSV against arsenic-induced hepatic bile acid accumulation and the consequent liver fibrosis. Notably, RSV intervention did not significantly alter the body weight or liver coefficient of rats (Figure S1), but it effectively reduced the arsenic concentration in the liver tissue. Assays of liver function and fibrosis-related indicators showed that RSV intervention led to a significant decrease in the activities of ALT and AST (Figure 2A–C). The hepatic levels of fibrosis markers, including α-SMA, COL-IV, LN, PIIINP, and HA, were remarkably lower in the RSV-treated group than those in the arsenic-only exposed group (Figure 2D,E). Histopathological observations further confirmed the efficacy of RSV in mitigating liver fibrosis. Compared with the arsenic-only exposed group, the RSV-treated group exhibited a marked reduction in collagen deposition in the hepatic vascular regions, and gradual restoration of the abnormal hepatic lobular architecture (Figure 2F). To explore whether the anti-fibrotic effect of RSV is associated with the regulation of bile acid accumulation, we further measured the levels of hepatic TBA and inflammatory cytokines after intervention. The results showed that RSV significantly abrogated arsenic-induced elevation of hepatic TBA levels and downregulated the expression of inflammatory cytokines IL-6, IL-1β, and TNF-α (Figure 2G,H). Taken together, these findings suggest that the protective effects of RSV against arsenic-induced liver fibrosis are linked to reduced hepatic bile acid accumulation and mitigated inflammatory responses.

### 2.3. Resveratrol Attenuates Arsenic-Induced Aberrations in the Hepatic Bile Acid Metabolic Profile

**Figure 3:**
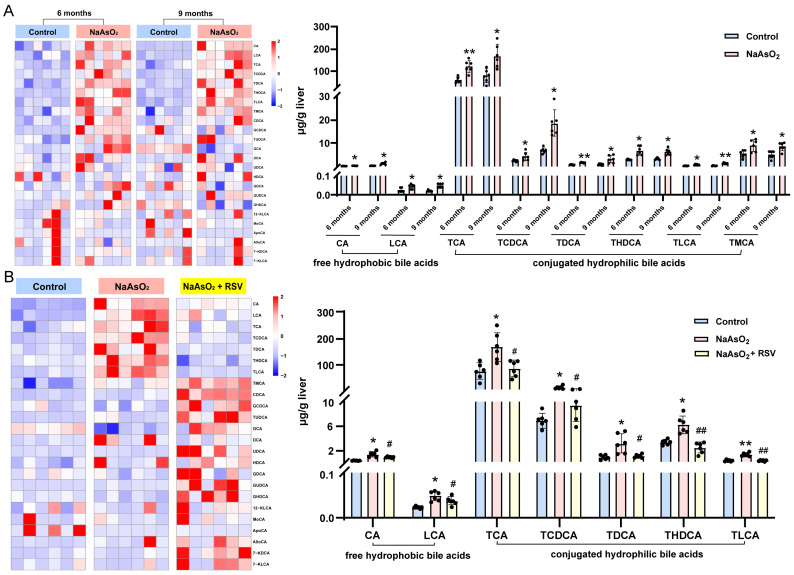
Resveratrol attenuates arsenic-induced aberrations in the hepatic bile acid metabolic profile. (**A**) Changes in hepatic bile acids in rats with arsenic-induced liver fibrosis. Heatmap showing the relative contents of 24 hepatic bile acid metabolites in individual rats after 6 and 9 months of arsenic exposure and their time-matched controls, with data standardized using Z-score normalization; bar plot showing the contents of bile acid metabolites that were significantly altered at both 6 and 9 months of arsenic exposure compared with the corresponding time-matched controls. (**B**) Effect of resveratrol on abnormal hepatic bile acids in rats with arsenic-induced liver fibrosis. Heatmap showing the relative contents of 24 hepatic bile acid metabolites in individual rats exposed to arsenic for 9 months, continuously exposed to arsenic and co-treated with resveratrol from month 6 to 9, and their time-matched controls, with data standardized using Z-score normalization; bar plot showing the contents of bile acid metabolites that were significantly different after resveratrol intervention compared with the arsenic-exposed group. All data were shown as mean ± SD (n = 6). Independent-samples *t*-test was used for all predefined pairwise comparisons. * *p* < 0.05, ** *p* < 0.001 vs. time-matched control; ^#^ *p* < 0.05, ^##^ *p* < 0.001 vs. NaAsO_2_.

The liver is the primary organ responsible for bile acid metabolism, and dysregulated bile acid metabolism is a key contributor to hepatic bile acid accumulation. Thus, we performed targeted bile acid metabolomics to investigate the effects of long-term arsenic exposure and RSV intervention on the hepatic bile acid metabolic profile in rats. The results showed that compared with the control group, hepatic levels of free hydrophobic bile acids cholic acid (CA) and lithocholic acid (LCA), as well as conjugated hydrophilic bile acids taurocholic acid (TCA), taurochenodeoxycholic acid (TCDCA), taurodeoxycholic acid (TDCA), taurohyodeoxycholic acid (THDCA), taurolithocholic acid (TLCA), and tauromuricholic acid (TMCA) were significantly increased in both the 6-month and 9-month arsenic exposure groups (Figure 3A and Table S1). RSV intervention effectively attenuated the arsenic-induced hepatic accumulation of free hydrophobic bile acids CA and LCA, and conjugated hydrophilic bile acids TCA, TCDCA, TDCA, THDCA, and TLCA (Figure 3B and Table S2). These findings suggest that long-term arsenic exposure leads to hepatic bile acid metabolic disorders characterized by increased free hydrophobic and partial conjugated hydrophilic bile acids, and RSV intervention improves this metabolic disturbance.

### 2.4. Resveratrol Alleviates Arsenic-Induced Impairments in Hepatic Bile Acid Phase II Detoxification and Excretion

**Figure 4:**
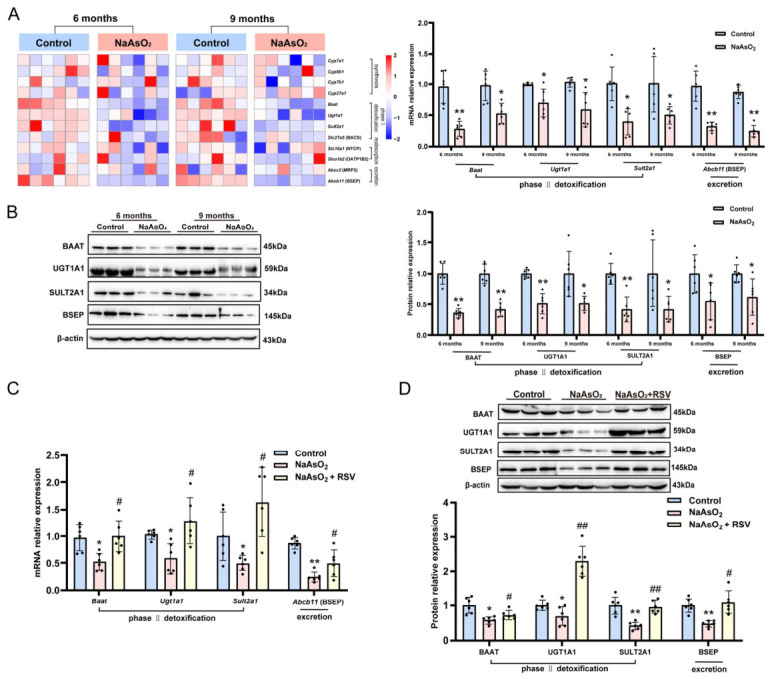
Resveratrol attenuates arsenic-induced inhibition of hepatic bile acid phase II detoxification enzyme and efflux protein expression. (**A**) mRNA levels of typical hepatic bile acid metabolism-related genes in rats with arsenic-induced liver fibrosis. Heatmap showing the relative expression levels of 12 hepatic bile acid metabolism-related genes in individual rats after 6 and 9 months of arsenic exposure and their time-matched controls; bar plot showing the mRNA expression levels of differentially expressed genes *Baat*, *Ugt1a1*, *Sult2a1*, and *Abcb11* (BSEP) that were significantly altered at both 6 and 9 months of arsenic exposure compared with the corresponding time-matched controls. (**B**) Hepatic protein levels of BAAT, UGT1A1, SULT2A1, and BSEP in rats exposed to arsenic for 6 and 9 months. (**C**) mRNA and (**D**) protein levels of *Baat*, *Ugt1a1*, *Sult2a1*, and *Abcb11* (BSEP) in the liver of rats exposed to arsenic for 9 months with resveratrol intervention. All data were shown as mean ± SD (n = 6). Independent-samples *t*-test was used for all predefined pairwise comparisons. * *p* < 0.05, ** *p* < 0.001 vs. time-matched control; ^#^ *p* < 0.05, ^##^ *p* < 0.001 vs. NaAsO_2_.

Based on the aforementioned hepatic bile acid metabolic profile results, we further detected bile acid metabolism-related gene expression to identify the key pathway of long-term arsenic-induced bile acid metabolic disorders and RSV’s intervention effect. Compared with the control group, 6-month and 9-month arsenic exposure significantly downregulated the hepatic mRNA expression of the phase II detoxification genes *Baat*, *Ugt1a1*, and *Sult2a1*, as well as the excretion gene *Abcb11* (BSEP) (Figure 4A). In contrast, no significant changes were observed in the mRNA levels of genes involved in bile acid synthesis, including *Cyp7a1*, *Cyp8b1*, *Cyp7b1*, and *Cyp27a1*, genes related to bile acid reabsorption, including *Slc10a1* (NTCP) and *Slco1b2* (OATP1B2), the phase II detoxification gene *Slc27a5* (BACS), or the excretion gene *Abcc2* (MRP2). Protein expression verification of these differentially expressed genes showed that arsenic exposure significantly reduced hepatic protein levels of the phase II detoxification genes BAAT, UGT1A1, SULT2A1, and the excretion gene BSEP (Figure 4B). Together with the observed accumulation of free hydrophobic and conjugated hydrophilic bile acids, these results demonstrate that long-term arsenic exposure disturbs bile acid metabolism, which is mainly associated with suppressed hepatic phase II detoxification and excretion pathways. Notably, RSV intervention significantly reversed arsenic-induced decreases in mRNA and protein expression of the phase II detoxification genes *Baat*, *Ugt1a1*, *Sult2a1*, and the excretion gene *Abcb11* (BSEP) (Figure 4C,D). Collectively, arsenic exposure inhibits the expression of key genes involved in phase II detoxification and excretion. These alterations are correlated with accumulation of free hydrophobic bile acids and certain conjugated hydrophilic bile acids. RSV treatment restores the expression of these genes and relieves arsenic-induced metabolic dysregulation.

### 2.5. Resveratrol Relieves Arsenic-Induced Hepatic Bile Acid Metabolism Dysfunction Associated with Restoring SIRT1/C/EBPα Axis Homeostasis

**Figure 5:**
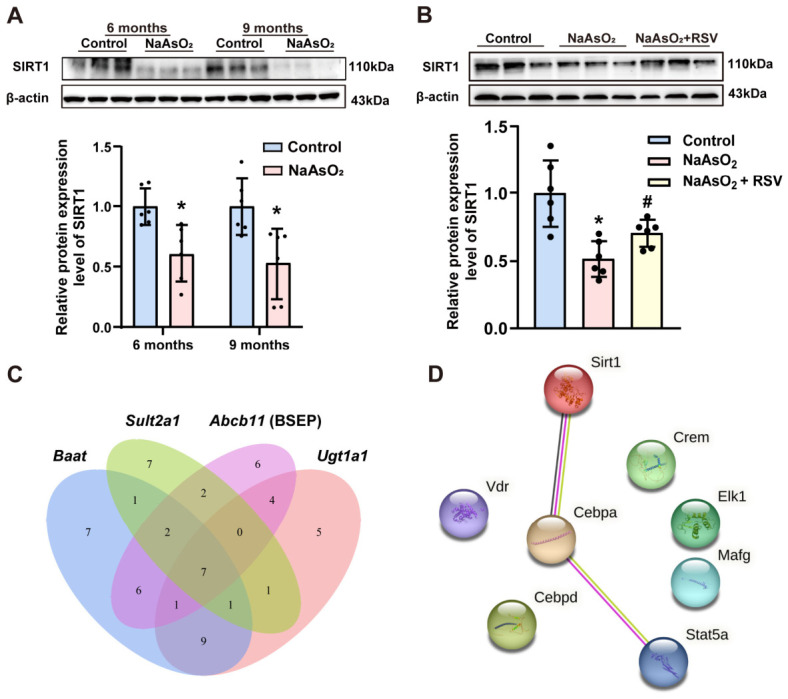
Resveratrol ameliorates arsenic-induced inhibition of liver SIRT1 expression. (**A**) Western blotting analysis of SIRT1 expression in the liver of rats exposed to arsenic for 6 and 9 months. (**B**) Western blotting analysis of SIRT1 expression in the liver of rats exposed to arsenic for 9 months with resveratrol intervention. (**C**) Venn diagram showing the number of transcription factors commonly regulating the four arsenic-responsive bile acid metabolism-related genes. (**D**) PPI network predicting the potential transcription factors interacting with SIRT1. All data were shown as mean ± SD (n = 6). Independent-samples *t*-test was used for all predefined pairwise comparisons. * *p* < 0.05 vs. time-matched control; ^#^ *p* < 0.05 vs. NaAsO_2_.

SIRT1 is a newly characterized regulator of bile acid metabolism and a well-established pharmacological target of RSV. We therefore explored the mechanisms underlying arsenic-induced impairments in hepatic bile acid phase II detoxification and excretion, as well as the protective action of RSV, with a specific focus on SIRT1. We first evaluated hepatic SIRT1 protein expression in rats and observed that 6-month and 9-month arsenic exposure markedly decreased SIRT1 levels compared with the control group (Figure 5A). RSV intervention effectively reversed arsenic-induced downregulation of SIRT1 (Figure 5B). Together with above findings, these results suggest that the ameliorative effects of RSV on arsenic-induced hepatic bile acid metabolic dysfunction may be linked to restored SIRT1 expression.

**Figure 6:**
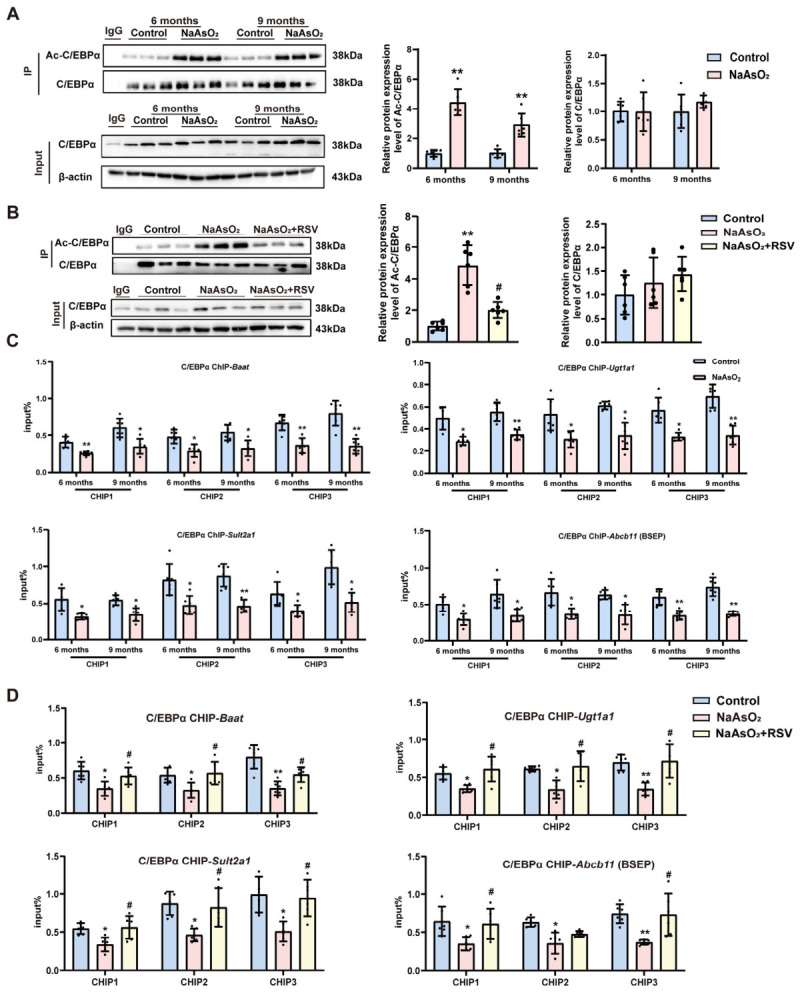
Resveratrol suppresses arsenic-induced hepatic C/EBPα acetylation to enhance its binding to bile acid phase II detoxification enzymes and efflux proteins. (**A**) Expression and acetylation levels of C/EBPα in the liver of rats exposed to arsenic for 6 and 9 months. (**B**) Expression and acetylation levels of C/EBPα in the liver of rats exposed to arsenic for 9 months with resveratrol intervention. (**C**) Enrichment levels of C/EBPα in the promoter regions of *Baat*, *Ugt1a1*, *Sult2a1*, and *Abcb11* (BSEP) in rat liver after 6 and 9 months of arsenic exposure. (**D**) Enrichment levels of C/EBPα in the promoter regions of *Baat*, *Ugt1a1*, *Sult2a1*, and *Abcb11* (BSEP) in rat liver exposed to arsenic for 9 months with resveratrol intervention. All data were shown as mean ± SD (n = 6). Independent-samples *t*-test was used for all predefined pairwise comparisons. * *p* < 0.05, ** *p* < 0.001 vs. time-matched control; ^#^ *p* < 0.05.

SIRT1 regulates gene expression by maintaining the acetylation homeostasis of transcription factors. We therefore used the PROMO database to predict potential transcription factors for the hepatic bile acid phase II detoxification genes *Baat*, *Ugt1a1*, *Sult2a1,* and the excretion gene *Abcb11* (BSEP). Candidate transcription factors conserved between humans and rats and expressed in the liver were further selected (Table S3) to identify downstream mediators through which SIRT1 modulates these target genes. Venn diagram analysis identified seven common transcription factors for the four genes, and protein–protein interaction analysis revealed the strongest interaction between CCAAT/enhancer-binding protein alpha (C/EBPα) and SIRT1 (Figure 5C,D; Table S4). We subsequently examined hepatic C/EBPα expression and acetylation status in rats. Compared with the control group, 6-month and 9-month arsenic exposure did not significantly alter total C/EBPα protein expression, but significantly increased its acetylation level (Figure 6A). This observation is consistent with the arsenic-mediated reduction in the deacetylase SIRT1. Further analysis in RSV treated rats demonstrated that RSV-mediated restoration of SIRT1 expression abolished arsenic-induced hyperacetylation of hepatic C/EBPα (Figure 6B). We then determined the enrichment of C/EBPα at the promoter regions of *Baat*, *Ugt1a1*, *Sult2a1,* and *Abcb11* (BESP). Hepatic C/EBPα binding to these gene promoters was significantly reduced following 6-month and 9-month arsenic exposure (Figure 6C), and this reduction was significantly ameliorated by RSV intervention (Figure 6D). Collectively, these results suggest that arsenic exposure suppresses SIRT1 expression, increases acetylation of the transcription factor C/EBPα and reduced recruitment of C/EBPα to the promoters of key genes responsible for hepatic phase II bile acid detoxification and excretion. These molecular alterations may relate to the suppressed expression of bile acid metabolic genes and hepatic bile acid metabolic disorders. The protective effects of RSV against arsenic-induced damage are linked to modulated SIRT1 expression and C/EBPα acetylation, alongside restored transcription of genes associated with hepatic bile acid metabolism.

## 3. Discussion

Arsenic is a well-recognized hepatotoxicant that induces progressive liver injury, ultimately leading to liver fibrosis and cirrhosis. Liver fibrosis, as a reversible intermediate stage of chronic liver disease, is a critical window for clinical intervention [33]. However, the lack of clear understanding of the early pathogenic mechanisms of arsenic-induced liver fibrosis has hindered the development of effective intervention strategies. This study identified that hepatic bile acid accumulation is an early event in the progression of arsenic-related liver fibrosis, and profiled the accompanying distinct bile acid metabolic changes and potential regulatory mechanism. We further demonstrated the potential link between resveratrol treatment and alleviated arsenic-induced liver fibrosis based on bile acid metabolism, offering novel experimental evidence for studying the pathology and management of arsenic-induced liver disorders.

Chronic liver fibrosis is driven by persistent liver injury, which triggers excessive extracellular matrix deposition mediated by activated HSCs. In recent years, accumulating evidence has suggested that bile acid metabolic dysregulation is closely associated with the initiation and progression of chronic liver diseases, serving as an early response to liver injury and a key mediator of inflammatory responses and HSC activation [17,34]. Under physiological conditions, bile acid homeostasis is precisely regulated by a coordinated network involving de novo synthesis, phase II detoxification, intestinal reabsorption, and biliary excretion [35,36]. Disruption of any link in this network can lead to intrahepatic bile acid accumulation, which in turn induces hepatocyte damage, inflammatory signaling activation, and the formation of a profibrotic microenvironment [37,38]. Although clinical studies have shown elevated serum bile acid levels in patients with arsenic-induced liver injury [7], the causal relationship between bile acid metabolic disturbance and arsenic-induced liver fibrosis remains unclear. Specifically, it is unknown whether hepatic bile acid accumulation acts as an early associated and plausible mediating event in the progression of arsenic-induced liver injury to fibrosis. To address this knowledge gap, we employed a time-dependent arsenic exposure model combined with targeted bile acid metabolomic analysis. Our results demonstrated that hepatic bile acid accumulation occurs at the early stage of arsenic exposure, accompanied by obvious inflammatory responses; with prolonged exposure, bile acid accumulation and inflammation are further exacerbated, followed by HSC activation and collagen deposition—the typical pathological features of liver fibrosis [15]. These findings suggest that hepatic bile acid accumulation is an early preceding event in arsenic-induced liver fibrosis, which reveals the temporal correlation of bile acid metabolic dysregulation in arsenic-induced liver injury and implies that bile acid accumulation may serve as a potential mediator participating in disease progression, rather than confirming its definite causal or driving role.

Notably, the accumulated bile acids in arsenic-exposed rat livers are mainly free hydrophobic bile acids (cholic acid, lithocholic acid) and partial conjugated hydrophilic bile acids (taurocholic acid, taurochenodeoxycholic acid, etc.). Previous studies have confirmed that free hydrophobic bile acids exhibit strong cytotoxicity, which can damage hepatocellular membranes, induce oxidative stress and apoptosis, activate pro-inflammatory signaling pathways, such as NOD-like receptor family, pyrin domain containing 3 (NLRP3) inflammasome, and directly promote HSC activation and proliferation [39]. Among these bile acids, LCA, a highly hydrophobic bile acid with the strongest toxicity that accumulates in the liver upon arsenic exposure, impairs the integrity of hepatic cell membranes and induces oxidative stress and apoptosis via reactive oxygen species (ROS) generation [40,41], thereby causing liver injury. LCA also triggers the NLRP3 inflammasome and induces the expression of inflammatory markers [42,43]. TCA can activate the Erk1/2 signaling pathway in hepatocytes and upregulate the expression of early growth response factor 1 (Egr-1), which in turn increases the production of inflammatory mediators and leads to liver injury [44]. TLCA induces cholestasis by impairing hepatobiliary exocytosis, inhibiting the insertion of transporters into the apical membrane and disrupting bile flow [45,46]. TCDCA and TDCA activate the NLRP3 inflammasome in a Ca^2+^ influx-dependent manner, resulting in excessive secretion of inflammatory cytokines [47]; this subsequently activates HSCs and contributes to the progression of liver tissue fibrosis [48]. In addition, Xie et al. [49] demonstrated that CA, LCA, TCA, TCDCA, TDCA, THDCA, TLCA, and TMCA all upregulated the expression levels of α-SMA and Transforming Growth Factor-beta (TGF-β) in LX-2 cells, thereby activating HSCs and promoting their proliferation. Intriguingly, all the above toxic bile acid subtypes were found to be significantly enriched in the liver upon arsenic exposure in our animal model. Combined with the well-established toxic and profibrotic properties of these bile acids reported in previous studies, our present findings further provide phenotypic evidence supporting that arsenic-triggered abnormal bile acid enrichment is closely linked to intrahepatic inflammatory response and subsequent HSC activation. Collectively, this work further highlights the important potential mediating role of bile acid metabolic dysregulation in the whole pathological cascade of arsenic-related liver damage and fibrotic progression.

To further elucidate the mechanisms of arsenic-induced hepatic bile acid accumulation, we focused on the key pathways regulating bile acid homeostasis, including synthesis, phase II detoxification, reabsorption, and excretion. Our results showed that arsenic exposure had no significant effect on the expression of genes related to bile acid synthesis (*Cyp7a1*, *Cyp8b1*, *Cyp7b1*, *Cyp27a1*) or reabsorption (*Slc10a1*, *Slco1b2*) but significantly downregulated the expression of phase II detoxification genes (*Baat*, *Ugt1a1*, *Sult2a1*) and the key biliary excretion transporter gene *Abcb11* (BSEP). These results indicate that impaired hepatic bile acid phase II detoxification and excretion may be the specific metabolic disorders responsible for arsenic-induced hepatic bile acid accumulation, rather than abnormal synthesis or reabsorption. Mechanistically, BAAT is responsible for the conjugation of free bile acids with taurine, which enhances the hydrophilicity of bile acids and facilitates their excretion [50]; *Ugt1a1* and *Sult2a1* mediate the glucuronidation and sulfation of hydrophobic bile acids, respectively, further promoting their detoxification and excretion [35,51,52]; BSEP is the core transporter responsible for the efflux of conjugated bile acids from hepatocytes into bile canaliculi [53,54]. The synergistic downregulation of these genes induced by arsenic exposure leads to a cascade of metabolic defects: reduced Baat expression impairs the conjugation of free bile acids, leading to the accumulation of toxic free bile acids; decreased *Ugt1a1* and *Sult2a1* expression inhibits the detoxification of hydrophobic bile acids, reducing their water solubility and excretion efficiency; and downregulated *Abcb11* (BSEP) expression directly blocks the main efflux pathway of conjugated bile acids, further exacerbating intrahepatic bile acid accumulation. Moreover, the accumulated toxic bile acids can further damage the hepatocellular canalicular membrane, inhibiting *Abcb11* (BSEP) function and forming a vicious cycle that perpetuates metabolic disturbance [55]. Under physiological conditions, cholesterol in the liver is synthesized into primary bile acids via two pathways: the classic pathway, initiated by CYP7A1 and regulated by CYP8B1, and the alternative pathway, initiated by CYP27A1 and regulated by CYP7B1. These primary bile acids are then detoxified through conjugation reactions under the action of phase II detoxifying enzymes, such as BAAT, SULT2A1, and UGT1A1, before being secreted into the bile canaliculi by hepatic efflux transporters (e.g., MRP2 and BSEP) [56,57]. Our findings confirm that arsenic specifically disrupts this detoxification–excretion axis, triggering a cascade reaction of “impaired conjugation and modification–detoxification disorder–blocked efflux pathway”, which induces the abnormal accumulation of free bile acids (CA, LCA) and tauro-conjugated bile acids (TCA, TCDCA, TDCA, THDCA, TLCA, TMCA) in the liver. These findings not only reveal the potential mechanism of arsenic-induced hepatic bile acid accumulation but also provide new experimental evidence for understanding arsenic-induced liver injury from the perspective of bile acid metabolic regulation, laying a foundation for the development of targeted intervention strategies. Notably, RSV intervention significantly restored the expression of the aforementioned phase II detoxification enzymes and efflux transporters; the increased expression of these detoxification enzymes could facilitate the sulfation, polyhydroxylation, or glycosylation of toxic bile acids, converting them into more hydrophilic substances for excretion [51,52], while the upregulation of efflux transporters further enhanced the efflux efficiency of bile acids [53], thereby effectively reducing the hepatotoxicity of bile acids.

To explore the upstream regulatory mechanism of arsenic-induced impairment in bile acid phase II detoxification and excretion, we focused on SIRT1, a NAD^+^-dependent deacetylase that plays a core role in maintaining hepatic metabolic homeostasis. SIRT1 regulates various physiological processes, including glucose and lipid metabolism, oxidative stress, and detoxification, by mediating the deacetylation of downstream target proteins [58,59,60]. Emerging evidence has shown that SIRT1 is a key regulator of bile acid homeostasis, and its dysregulation can directly lead to hepatic bile acid accumulation and liver injury [61]. Although previous studies have reported that arsenic exposure inhibits SIRT1 expression, the role of SIRT1 in arsenic-induced bile acid metabolic disturbance remains unclear. In this study, we found that arsenic exposure significantly downregulated hepatic SIRT1 protein expression, while RSV intervention effectively restored SIRT1 levels. Given that SIRT1 exerts its regulatory effects mainly through modulating the acetylation status of transcription factors, we predicted the common transcription factors of *Baat*, *Ugt1a1*, *Sult2a1*, and *Abcb11* (BSEP) using bioinformatic tools (PROMO database), and identified C/EBPα as a potential key transcription factor that interacts with SIRT1. C/EBPα is highly expressed in the liver and participates in various hepatic physiological and pathological processes, including hepatocyte proliferation and differentiation, as well as the development of chronic hepatitis, hepatic steatosis [62,63], liver fibrosis [64], and hepatocellular carcinoma [65,66], but its regulatory role in bile acid metabolism has rarely been reported. Our results showed that arsenic exposure did not affect the total protein expression of C/EBPα, but significantly increased its acetylation level and reduced its enrichment at the promoter regions of *Baat*, *Ugt1a1*, *Sult2a1*, and *Abcb11* (BSEP). Previous studies have confirmed that acetylation modification of C/EBPα can directly regulate its transcriptional binding activity to the promoter regions of target genes by altering the spatial conformation of its DNA-binding domain and its DNA-binding affinity [67]. We therefore conclude that arsenic exposure suppresses SIRT1 expression, accompanied by abnormal hyperacetylation of C/EBPα and reduced transcriptional activity of bile acid metabolism-related genes. These molecular alterations may relate to impression of hepatic phase II bile acid detoxification and excretion. This study elucidates a novel potential mechanism underlying arsenic-induced bile acid metabolic disorder. Aberrant SIRT1-mediated acetylation modification of C/EBPα may act as a potential upstream molecular event mediating arsenic-induced bile acid accumulation, and provide supplementary evidence for exploring the pathogenesis of bile acid retention-related liver diseases within the SIRT1-regulated metabolic pathway.

RSV, a natural polyphenol and classic SIRT1 activator, exerts well-documented anti-inflammatory, antioxidant, and hepatoprotective effects. Previous studies demonstrated that RSV attenuates liver fibrosis induced by CCl_4_ or inorganic mercury mainly by inhibiting nuclear factor-κB (NF-κB)-mediated inflammation or activating the SIRT1/ peroxisome proliferator-activated receptor gamma coactivator 1-alpha (PGC-1α) pathway, thereby suppressing oxidative stress and HSC activation [68,69,70]. However, it remains unclear whether resveratrol (RSV) can ameliorate arsenic-induced liver fibrosis via regulating hepatic bile acid metabolism. Our results demonstrate that RSV intervention alleviates the intrahepatic accumulation of hydrophobic bile acids caused by arsenic exposure, along with subsequent hepatic inflammation and fibrosis. Such hepatoprotective effects may be associated with the reversal of arsenic-mediated SIRT1 downregulation, abnormal elevation of C/EBPα acetylation, as well as the recovery of impaired binding between C/EBPα and the promoters of hepatic phase II bile acid detoxification and efflux genes [*Baat*, *Ugt1a1*, *Sult2a1*, *Abcb11* (BSEP)], and the restoration of the expression of these genes. These findings suggest a plausible regulatory mechanism. This study reveals a previously unrecognized association between the SIRT1/C/EBPα acetylation axis, disordered hepatic bile acid metabolism, and arsenic-induced liver fibrosis.

Importantly, our data confirmed that RSV reduces hepatic arsenic accumulation in arsenic-exposed rats, indicating that RSV-mediated hepatoprotection is not merely dependent on the regulation of the SIRT1/C/EBPα signaling pathway. RSV may lower hepatic arsenic burden by interfering with intestinal arsenic absorption and promoting systemic arsenic metabolism and excretion. In the current in vivo experimental system, the SIRT1/C/EBPα pathway-modulating effect and arsenic-clearing effect of RSV occur simultaneously and cannot be fully disentangled. Collectively, the results indicate that RSV may act against arsenic-induced liver fibrosis through two pathways. On the one hand, it may regulate the SIRT1/C/EBPα axis to restore bile acid homeostasis disrupted by arsenic. On the other hand, it may reduce arsenic accumulation in the liver and thereby alleviate arsenic-induced liver damage.

These findings have two important implications. First, these findings preliminarily reveal the intervention effect and potential molecular mechanism of RSV against arsenic-induced liver fibrosis, providing a natural compound strategy and experimental basis for the early intervention of arsenic-induced liver fibrosis; second, they suggest that RSV may be a promising candidate agent for the early intervention of various bile acid accumulation-related liver diseases, expanding its potential clinical application value. Accumulating evidence has also demonstrated that RSV can regulate the body’s bile acid metabolic homeostasis by modulating bile acid synthesis, transport, and enterohepatic circulation, thereby ameliorating pathological injuries associated with abnormal hepatic bile acid accumulation [71,72,73], which further supports our findings on the protective role of RSV in arsenic-induced liver fibrosis.

While this study provides novel insights into the mechanism and intervention of arsenic-induced liver fibrosis by identifying potential mediators and intervention targets, it has several limitations that need to be addressed. First, rigorous mechanistic and functional validation experiments are lacking. Specifically, we did not conduct SIRT1/C/EBPα functional validation (including SIRT1 inhibition/overexpression, C/EBPα gain- and loss-of-function, and rescue experiments) or acetylation-specific mechanistic validation (e.g., experiments targeting C/EBPα acetylation sites). This restricts the causal interpretation of the SIRT1/C/EBPα-dependent mechanism, which requires further verification. Second, the study did not set an independent RSV-only control group, making it impossible to quantitatively distinguish the direct regulatory effects of RSV on signaling pathways from its indirect effects via arsenic elimination in the body. In follow-up studies, we will perform in vivo and in vitro verification to clarify the specific mechanism by which RSV regulates arsenic absorption, metabolism, and excretion, and further differentiate its dual protective modes. Additionally, we need to explore the dose-response relationship of RSV intervention, clarify its optimal therapeutic dose and long-term application safety, to provide solid experimental evidence for clinical translation. Third, although we confirmed hepatic bile acid accumulation as an early event of arsenic-induced liver fibrosis and explored related metabolic mechanisms, we only detected hepatic bile acid profiles and the expression of related genes and proteins. Functional assays (e.g., serum/fecal bile acid detection, bile flow measurement, and transporter localization) to verify bile acid transport and detoxification function were not performed. Moreover, the roles of intestinal flora and the gut-liver axis in this pathological process remain unclear, which requires further research to fill these gaps. Fourth, while the study preliminarily clarified the potential mechanism of arsenic-induced liver fibrosis and verified the overall intervention efficacy of RSV, the sample size is limited. Expanding the sample size in subsequent studies will improve statistical power and the robustness of the findings, which will be used to further validate both the mechanistic conclusions of arsenic-induced liver fibrosis and the hepatoprotective effects of RSV. We also acknowledge another limitation of this study. Complete blinding was not achieved during Western blot experiments owing to the requirement of group-linked sample numbering for sample management and data comparison. To mitigate potential bias, all protein band quantification was conducted using standardized software settings for automated analysis. Even so, the absence of full blinding in Western blot assays should be noted as a methodological limitation.

## 4. Materials and Methods

### 4.1. Animals and Treatments

Thirty specific pathogen-free (SPF) healthy Sprague-Dawley rats with an initial body weight of 80–120 g were purchased from Liaoning Changsheng Biotechnology Co., Ltd., Benxi, China, (Animal Production License No.: SCXK (Liao) 2020-0001). All rats were housed in an SPF animal facility under controlled conditions (temperature, 20–25 °C; relative humidity, 50–60%; 12 h light/dark cycle), with 6 rats per group (1:1 male-to-female ratio). Rats were individually caged with free access to standard chow and drinking water. After a 1-week adaptive feeding period, the rats were randomly divided into five groups: 6-month control group, 6-month sodium arsenite (NaAsO_2_) group, 9-month control group, 9-month NaAsO_2_ group, and NaAsO_2_ + RSV group. According to our previously established protocol [74,75], rats in the control groups were given deionized water via gavage. For arsenic-exposed groups, 2.00 g/L NaAsO_2_ solution was administered by gavage at a dose of 10.0 mg/kg·bw (1/4 LD_50_ of NaAsO_2_ in rats, equivalent to 1555 μg/L elemental arsenic in groundwater which is consistent with the levels reported in some high arsenic-contaminated areas) once daily for 6 consecutive days per week, with exposure lasting for 6 or 9 months, respectively. This intragastric gavage mimics human oral exposure via drinking water and diet, and the 6–9-month exposure duration (equivalent to 15–25.2 years of human exposure) simulate long-term arsenic exposure in humans. Rats in the NaAsO_2_ + RSV group first received NaAsO_2_ treatment at the aforementioned dose and regimen for 6 months, a time point at which hepatic bile acid accumulation was observed without detectable liver fibrosis phenotypes. Subsequently, these rats were gavaged with 10.0 mg/kg·bw NaAsO_2_ daily, followed by gavage administration of 20 mg/kg bw RSV at a 4-h interval; this combined treatment was performed for 6 consecutive days per week until the 9-month time point. At the end of the treatment period, all rats were fasted for 12 h and their body weights were recorded. After anesthesia with 1% pentobarbital sodium, liver tissues were harvested and weighed immediately. A portion of the liver tissue was fixed in 4% paraformaldehyde for histopathological examination, and the remaining tissue was snap-frozen and stored at −80 °C for subsequent experimental assays. This animal study protocol was approved by the Institutional Animal Care and Use Committee of Guizhou Medical University (Approval Code: 1702146; Approval Date: 8 March 2017).

### 4.2. Arsenic Concentration Assay

Approximately 10 mg of liver tissue was immersed in a mixture of concentrated HNO_3_ and 30% H_2_O_2_ for 2 h. Then, the liver tissue was transferred to the microwave digestion system (Milestone ETH, Bergamo, Italy) and digested according to the following procedure: 120 °C for 5 min, 180 °C for 10 min, and 190 °C for 15 min. The arsenic concentration in the liver tissue was measured using inductively coupled plasma mass spectrometry (ICP-MS, NexION 2000, PerkinElmer Inc., Waltham, MA, USA), and the arsenic concentration in the liver tissue was quantitatively calculated using a standard curve, and normalized by the weight of the digested liver tissue.

### 4.3. Liver Function Assay

Approximately 30 mg of liver tissue was homogenized at a ratio of 1:9 (*w*/*v*) in PBS. The activities of ALT and AST in the liver tissue were detected according to the instructions of the ALT and AST kits (C009-2-1 and C010-2-1, Nanjing Jiancheng Institute of Bioengineering, Nanjing, China). The linear detection range for both ALT and AST were 0–200 Karman units.

### 4.4. Immunohistochemistry

Paraffin sections of liver tissue were routinely dehydrated and then placed in a citric acid hydrochloric acid buffer and heated for antigen repair. 10% goat serum was blocked for 30 min and then incubated overnight with α-SMA antibody (1:2000 dilution, ab124964, Abcam, Cambridge, UK) at 4 °C. The next day, it was incubated with the secondary antibody at room temperature for 1 h. The DAB stain was applied for 5 min and then re-stained with hematoxylin for another 5 min. Finally, the sheet was sealed with neutral gum after dehydration by soaking in 95% ethanol and transparent treatment with xylene. Five field of view images were collected in each section using an optical microscope (Nikon, Tokoyo, Japan), and the average optical density value of the positive expression region of α-SMA was calculated using Image J (Version 1.54f).

### 4.5. Histopathological Assay

Liver tissues fixed with 4% paraformaldehyde were subjected to routine dehydration and paraffin embedding, followed by sectioning with a microtome. The sections were mounted on glass slides, incubated in a 65 °C oven for 2 h, and then subjected to subsequent staining procedures. Hematoxylin–eosin staining was performed to observe the general pathological changes of liver tissue. Masson staining was specifically used to visualize and quantify collagen deposition in liver tissue. Image J (Version 1.54f) was employed to calculate the collagen volume fraction (expressed as a percentage) for the objective assessment of total collagen fiber content in liver tissue, with five random fields of view selected per animal and their mean value adopted for subsequent analysis. In addition, Sirius Red staining was applied to further evaluate the content of different types of collagen fibers. Under polarized light microscopy, type I collagen fibers exhibited red birefringence, while type III collagen fibers showed green birefringence. ImageJ software was used to quantify the ratio of type I to type III collagen fibers, with three random fields of view selected per animal and their mean value adopted for subsequent analysis.

### 4.6. Enzyme-Linked Immunosorbent Assay

Briefly, 50 mg of liver tissue was homogenized in 9 volumes of physiological saline at a ratio of 1:9 (*w*/*v*). After centrifugation at 2500 rpm for 20 min, the supernatant was collected for subsequent analysis. Hepatic fibrosis markers were measured using commercial ELISA kits following the manufacturer’s instructions. COL-IV was determined using a rat COL IV ELISA kit (ml058987, mlbio, Shanghai, China), with a detection range of 3.13–200 ng/mL, a sensitivity of 1.56 ng/mL, intra-assay CV < 10%, and inter-assay CV < 15%. LN was detected with a rat LN ELISA kit (ml868874, mlbio, Shanghai, China), with a detection range of 31.25–2000 ng/mL, a sensitivity of 15.63 ng/mL, intra-assay CV < 6%, and inter-assay CV < 8%. PIIINP was quantified using a rat PIIINP ELISA kit (ml028351, mlbio, Shanghai, China), with a detection range of 0.312–20 ng/mL, a sensitivity of 0.16 ng/mL, intra-assay CV < 8.4%, and inter-assay CV < 8.2%. HA was measured using a rat HA ELISA kit (CSB-E08120r, CUSABIO, Wuhan, China), with a detection range of 4.69–300 ng/mL, a sensitivity of 1.17 ng/mL, intra-assay CV < 8%, and inter-assay CV < 10%. Before detection, the supernatants were diluted 1:200 for COL-IV, 1:20 for LN, 1:600 for PIIINP, and 1:10 for HA. The hepatic levels of inflammatory cytokines, including IL-1β, IL-6, and TNF-α, were measured using corresponding ELISA kits (H002-1-2, H007-1-2, and H052-1-2; Nanjing Jiancheng Institute of Bioengineering, Nanjing, China) following the manufacturer’s protocols. For IL-1β, the detection range was 0.5–200 ng/L, sensitivity was 0.5 ng/L, intra-assay CV < 10%, and inter-assay CV < 12%. For IL-6, the detection range was 2–600 ng/L, sensitivity was 2 ng/L, intra-assay CV < 10%, and inter-assay CV < 12%. For TNF-α, the detection range was 5–1000 ng/L, sensitivity was 5 ng/L, intra-assay CV < 10%, and inter-assay CV < 12%, and the supernatant was used directly for detection. No further dilution of the supernatant was required for the detection of IL-1β, IL-6, and TNF-α. The concentrations of COL-IV, LN, PIIINP, HA, IL-1β, IL-6, and TNF-α in liver tissue were calculated from the standard curves and normalized by liver tissue weight.

### 4.7. Total Bile Acid Level Assay

TBA levels in liver tissue were measured using a Total Bile Acid Kit (E003-2-1, Nanjing Jiancheng Bioengineering Institute, Nanjing, China). The linear range was 0–180 μmol/L, intra-assay CV ≤ 5.0%, and inter-assay relative range ≤ 10.0%. Briefly, 50 mg of liver tissue was homogenized in 9 volumes of PBS (1:9 *w*/*v*), and the supernatant was diluted 1:5 before TBA detection. A total of 3 μL of liver supernatant was used for the enzymatic cycling reaction. The absorbance values of each measurement were read using an enzyme reader at a wavelength of 405 nm and were recorded as A0. After another 3 min of incubation, the absorbance values of each measurement were read again and recorded as A1. ΔA = A1 − A0. According to the formula: TBA (μmol/L) = C standard × (ΔA measurement/ΔA standard), the TBA levels of each sample were calculated.

### 4.8. Bile Acid Metabolism Profile Assay

A total of 30 mg liver tissue was homogenized with 100 μL deionized water. Amounts of 500 μL pre-cooled methanol and 10 μL of 200 ng/mL mixed internal standard were added sequentially, relevant information on internal standards is provided in Table S5. The mixture was vortexed for 60 s, sonicated at low temperature for 30 min twice, and incubated at −20 °C for 1 h to precipitate proteins. It was then centrifuged at 12,000 rpm and 4 °C for 20 min, and the supernatant was collected for subsequent analysis. The supernatant was separated on an Agilent 1290 Infinity LC UHPLC system. Samples were kept in a 4 °C autosampler with the column temperature at 45 °C. Mobile phase A was 0.1% formic acid aqueous solution and mobile phase B was pure methanol, with a flow rate of 250 μL/min and injection volume of 2 μL. The UHPLC gradient was 0–7 min, phase B linearly increased from 50% to 70%; 7–15 min, 70% to 90%; 15–17 min, maintained at 90%; 17–17.1 min, 90% to 60%; 17.1–20 min, maintained at 60%. QC samples were inserted at regular intervals in the sample queue to monitor system stability and repeatability. A standard mixture of target substances was included for chromatographic retention time correction. Mass spectrometric analysis was performed in negative ion mode using a 5500 QTRAP mass spectrometer (SCIEX). Electrospray Ionization (ESI) source conditions: source temperature 550 °C; Gas1 55; Gas2 55; CUR 40; ISVF−4500 V. Multiple Reaction Monitoring (MRM) mode was used to detect target ion pairs. All experimental samples were detected uniformly in a single experimental batch. Peak areas and retention times were acquired via MultiQuant 3.0.2. Metabolites were identified by retention time matching with reference standards. Using isotope internal standardization, serial standard curves were fitted by peak area ratios of analytes to internal standards. Sample concentrations were calculated, and final hepatic bile acid contents were corrected by initial sample weight. Curve parameters and linear ranges are shown in Table S6. Spiked recoveries were 85–115%, and RSDs of all QC samples were below 10%, meeting the criterion of <30%. The RSD distribution is presented in Figure S2.

### 4.9. Real-Time qPCR

Using the Prime Script TM RT reagent kit (Thermo Fisher, Waltham, MA, USA), total RNA isolated from liver tissues was reverse-transcribed. Real-time fluorescent quantitative polymerase chain reaction (qPCR) was performed using the TB Green Premix Ex Taq™ II Kit (Takara Bio, Tokyo, Japan), and the mRNA expression levels were calculated by the 2^−ΔΔCt^ method. The primer sequences are detailed in Table S7.

### 4.10. Western Blotting

Liver tissue (40 mg) was harvested from each rat and homogenized on ice in lysis buffer supplemented with protease and phosphatase inhibitors (Beyotime Biotechnology, Shanghai, China) to maintain protein integrity. The protein concentration of each tissue lysate was quantified using a BCA protein assay kit (Solarbio, Beijing, China).

Based on the molecular weight of the target protein, gels with appropriate concentrations were prepared for electrophoresis. Each sample was loaded with 30 μg protein. The initial electrophoresis voltage was set at 60 V; after the samples migrated through the stacking gel, the voltage was adjusted to 90 V and further increased to 120 V when the samples migrated halfway through the separating gel. Subsequently, proteins were transferred onto an NC membrane (Bio Trace, Bothell, WA, USA). The transfer current and duration were adjusted according to the molecular weight of target proteins, with the routine condition set at 300 mA for 60 min. The membrane was blocked with 5% skimmed milk for 2 h, then incubated overnight with primary antibodies against SIRT1 mouse mAb (1:1000 dilution, ab110304, Abcam, Cambridge, UK), C/EBPα rabbit mAb (1:2000 dilution, ab317442, Abcam, Cambridge, UK), UGT1A1 rabbit mAb (1:1000 dilution, ab194697, Abcam, Cambridge, UK), SULT2A1 rabbit mAb (1:1000 dilution, ab194113, Abcam, Cambridge, UK), and BSEP rabbit mAb (1:1000 dilution, ab155421, Abcam, Cambridge, UK), BAAT rabbit mAb (1:500 dilution, PA5-96744, Invitrogen, Waltham, MA, USA), β-actin rabbit mAb (1:10000 dilution, 20536-1-AP, Protein Tech, Wuhan, China). The secondary antibody Goat Anti-Rabbit IgG (H + L) HRP (1:8000 dilution, SA00001-2, Protein Tech, Wuhan, China) or Goat Anti-Mouse IgG (H + L) HRP (1:8000 dilution, SA00001-1, Protein Tech, Wuhan, China) was incubated at room temperature for 1 h the next day. Protein bands were developed with an ultra-sensitive chemiluminescence kit (Beyotime Biotechnology, Shanghai, China), and band signals were captured by a gel imaging system (Bio-Rad, Hercules, CA, USA) with exposure parameters set as follows: automatic exposure mode, graded manual exposure times ranging from 10 s to 5 min were sequentially collected to screen moderate signal bands without overexposure or underexposure. The gray values of target protein bands were quantified via ImageJ software. β-actin was used as an internal reference protein to normalize the expression of target proteins. After normalization to β-actin, the relative protein expression levels were determined by calibration against the mean value of the control group.

### 4.11. Transcription Factor Prediction and Analysis

The promoter sequences of hepatic bile acid metabolism-related genes *Baat*, *Ugt1a1*, *Sult2a1*, and *Abcb11* (BSEP) were retrieved and verified using the UCSC database. The PROMO database was used to predict the potential transcription factors (TFs) that bind to the promoter regions of each of these four genes separately. To ensure cross-species conservation and liver relevance, homologous genes between humans and rats that are expressed in the liver were screened via the NCBI database. Common transcription factors among the four TF sets were then identified by Venn diagram intersection analysis. SIRT1 and the aforementioned common transcription factors were uploaded to the STRING database to construct a protein–protein interaction (PPI) network, aiming to predict and characterize their potential interactions.

### 4.12. Immunoprecipitation

To approximately 30 mg liver tissue, 3 titanium dioxide grinding beads and lysis solution (with deacetylase) were added, grinded via homogenizer, and centrifuged supernatant. A small portion of the supernatant was reserved as the input sample for subsequent input normalization. This experiment was performed using the Pierce™ Classic Magnetic IP/Co-IP Kit (Thermo Fisher Scientific, 88804, Waltham, MA, USA). Normal rabbit IgG (Cell Signaling Technology, 2729S, Danvers, MA, USA) was used as the negative control. This antibody lacks specific antigen-binding activity and was applied to exclude non-specific binding. It was used at the same dosage (2 µg) as the primary antibody against the target protein. C/EBPα antibody (2 µg, ab317442, Abcam, Cambridge, UK) was incubated with resuspended Protein A+G magnetic beads on a rotary shaker at room temperature for 1 h, beads were washed with TBS. Sample was added to beads, incubated at 4 °C overnight (inversion shaker), and beads washed. SDS-PAGE loading buffer was added, heated at 95 °C for 5 min to get protein sample. Then, Western Blotting proceeded as usual. The C/EBPα antibody (ab317442, Abcam, Cambridge, UK) used for immunoprecipitation has been verified by the manufacturer to be applicable to immunoprecipitation assays via Western blotting and dot blot analyses. The relative enrichment level of the target protein was quantified by normalizing the gray band value of the target protein in immunoprecipitation fractions to that in the corresponding input samples. Meanwhile, background signals derived from the IgG control group were subtracted to obtain the specific binding level. Six independent liver tissue samples derived from six individual rats were set as biological replicates in each group, and the whole IP experiment was performed twice independently to guarantee experimental reproducibility.

### 4.13. Chromatin Immunoprecipitation–Quantitative Polymerase Chain Reaction Assay (ChIP-qPCR Assay)

ChIP-qPCR assays were conducted with the SimpleChIP^®^ Enzymatic Chromatin IP Kit (Cell Signaling Technology, Danvers, MA, USA) to detect C/EBPα enrichment in the promoters of *Baat*, *Ugt1a1*, *Sult2a1*, and *Abcb11* (BSEP) genes. The C/EBPα antibody (ab317442, Abcam, Cambridge, UK) used for ChIP has been verified by the manufacturer to be applicable to immunoprecipitation assays via Western blotting and dot blot analyses. Approximately 50 mg liver tissue was minced; protease inhibitor and 37% formaldehyde were added sequentially. Shaken at room temperature for 20 min (protein-DNA cross-linking), it was incubated with glycine for 5 min to quench fixation. A single-cell suspension was prepared per tissue supernatant protocol, centrifuged, and treated with micrococcal nuclease at 37 °C for 20 min; EDTA terminated digestion. After digestion and subsequent sonication, the fragmented chromatin was verified by 1.5% agarose gel electrophoresis, showing a DNA fragment size range of 150–900 bp, which is optimal for ChIP-qPCR. After 10 min ChIP buffer incubation, the sample was sonicated, and centrifuged supernatant was cross-linked chromatin. Take 50 μL chromatin, add 100 μL nuclease-free water, NaCl and RNase A, vortex, incubate at 37 °C for 30 min. Proteinase K was added, incubated at 65 °C for 2 h, then column-purify DNA was spun and concentration determined. Equal-mass chromatin was adjusted to 500 μL with ChIP buffer. A total of 10 μL of this chromatin mixture was reserved as the 2% input control for subsequent input normalization to correct for variations in initial chromatin amount and DNA recovery efficiency. The C/EBPα antibody (2 µg, ab317442, Abcam, Cambridge, UK)—the target antibody for detecting C/EBPα-chromatin binding—was used in parallel with normal rabbit IgG (2 µg, provided in the SimpleChIP^®^ Enzymatic Chromatin IP Kit), which served as the negative control. The isotype-matched normal rabbit IgG lacks specific DNA-binding capacity and was used to subtract non-specific background enrichment. The C/EBPα antibody was added to one aliquot of the 500 μL chromatin mixture, while the normal rabbit IgG was added to another separate aliquot; both tubes were sealed and rotated gently at 4 °C overnight to allow specific (for C/EBPα antibody) or non-specific (for IgG control) antibody-chromatin binding. Next, 30 μL ChIP-grade Protein G beads was added for 2-h 4 °C rotation. After magnetic separation and low/high-salt washes, 150 μL ChIP buffer was added for 30-min 37 °C incubation. Supernatant was collected, mixed with NaCl and proteinase K (65 °C for 2 h), then combined with DNA binding buffer for spin column purification (washed, eluted) to obtain purified DNA. qPCR was performed using the TB Green Premix Ex Taq™ II Kit (Takara Bio, Tokyo, Japan), and the antibody enrichment efficiency was calculated by the Percent Input method (% Input = 2^(−ΔCt[normalized ChIP]) × 100%). Each group included liver tissue samples from six independent rats as biological replicates; each sample was examined in two technical replicate qPCR assays, and the mean value of the two technical replicates was used for data analysis. Primer sequences are in Table S8. Fold enrichment was calculated as the ratio of relative enrichment in the C/EBPα antibody group to that in the IgG control group; a fold enrichment ≥ 2 was defined as specific binding of C/EBPα to the target gene promoter, and signals close to IgG background (fold enrichment < 2.0) were regarded as non-specific. IgG control and fold enrichment results are in Figure S3.

### 4.14. Blinding Protocols

For all experimental indicators except animal model establishment and Western blot experiments, an independent researcher not involved in experimental grouping or intervention was responsible for sample coding. Each sample was assigned a unique code, with the corresponding relationship between the code and experimental group strictly kept confidential until all experimental detections and analyses were completed. Throughout the experimental process, investigators responsible for histological staining, immunohistochemical detection, image acquisition, quantitative analysis, bile acid metabolic profiling, and the determination of related gene expression levels remained completely unaware of sample grouping information, using only the unique codes to identify samples and thereby minimizing potential observer bias associated with prior knowledge of sample groups. Blinding was not applicable during animal model establishment, as explicit group division was required to guarantee accurate model construction. For Western blot analysis, group-linked sample numbering was mandatory for sample loading, intergroup comparison and sample tracking. Although we applied unified fixed parameters in ImageJ for automated band quantification to reduce subjective bias, full blinding could not be implemented in this assay.

### 4.15. Statistical Analysis

SPSS 23.0 was used for statistical analysis of experimental data. A total of 30 rats were used in this study. The sample size in the present study was determined based on the primary hepatic fibrosis-related outcomes, including COL IV, PIIINP, LN, HA and α-SMA, according to previous arsenic-induced liver fibrosis studies [75]. Analyses of molecular and metabolomic data in this study should therefore be interpreted as supportive evidence for mechanistic exploration. The Grubbs’ test was performed to screen potential outliers during data sorting, and no valid outliers were detected in all datasets. The Shapiro–Wilk test was used to test the normal distribution of original data, and Levene’s test was applied to assess variance homogeneity. All measured data in this study satisfied normal distribution and were presented as mean ± standard deviation (mean ± SD). All inter-group comparisons were pre-specified pairwise comparisons. Specifically, independent-samples *t*-test was used to compare the 6-month arsenic exposure group with its time-matched control group, the 9-month arsenic exposure group with its matched control group, and the 9-month arsenic exposure group with the resveratrol intervention group. Differences with *p* < 0.05 were considered statistically significant. Detailed statistical data of all measured endpoints are provided in Tables S9–S14.

## 5. Conclusions

In summary, hepatic bile acid accumulation may be an early critical event in arsenic-induced liver fibrosis, and impaired phase II detoxification and excretion may represent the specific metabolic disorder underlying such accumulation. SIRT1, C/EBPα acetylation, and bile acid metabolism are correlated with arsenic-induced liver fibrosis. Arsenic exposure inhibits SIRT1 expression and induces excessive acetylation of the transcription factor C/EBPα, which may potentially impair the transcriptional activity of C/EBPα toward the bile acid metabolism genes *Baat*, *Ugt1a1*, *Sult2a1*, and *Abcb11* (BSEP), and ultimately suppressing hepatic bile acid phase II detoxification and efflux. Importantly, resveratrol may restore SIRT1 expression, normalize C/EBPα acetylation and bile acid homeostasis, and reduce hepatic arsenic accumulation, thereby alleviating liver fibrosis. These findings may offer potential clues for early monitoring and intervention of arsenic-induced liver fibrosis.

## Data Availability

The original contributions presented in this study are included in the article/Supplementary Materials. Further inquiries can be directed to the corresponding author.

## Supplementary Materials

The following supporting information can be downloaded at: https://www.mdpi.com/article/10.3390/ijms27115123/s1.
