# Supplementary material for: Resveratrol Alleviates Arsenic-Induced Liver Fibrosis in Rats by Correcting SIRT1-Mediated Disorder of Hepatic Bile Acid Metabolism
Source: Int J Mol Sci. 2026 Jun 5;27(11):5123. doi: 10.3390/ijms27115123 (PMC13257504; doi:10.3390/ijms27115123)
Supplement: Supplementary file 1 [file ijms-27-05123-s001.zip › Figures S1–S3.pdf]

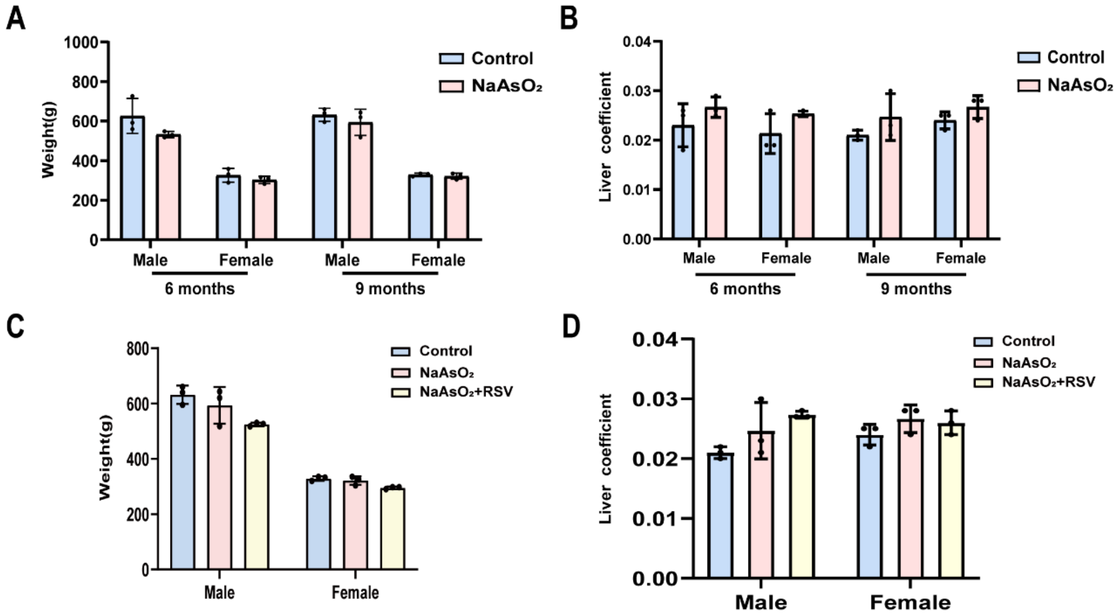

Figure S1. Body weight and liver coefficient of rats in each group. (A) Body weight of rats exposed to arsenic at different time points. (B) Liver coefficient of rats exposed to arsenic at different time points. (C) Body weight of rats with resveratrol intervention. (D) Liver coefficient of rats with resveratrol intervention. Data were obtained from rats with 6- and 9-month arsenic exposure and their time-matched controls. All data were shown as mean ± SD (n = 6). Independent-samples t-test was used for all predefined pairwise comparisons.

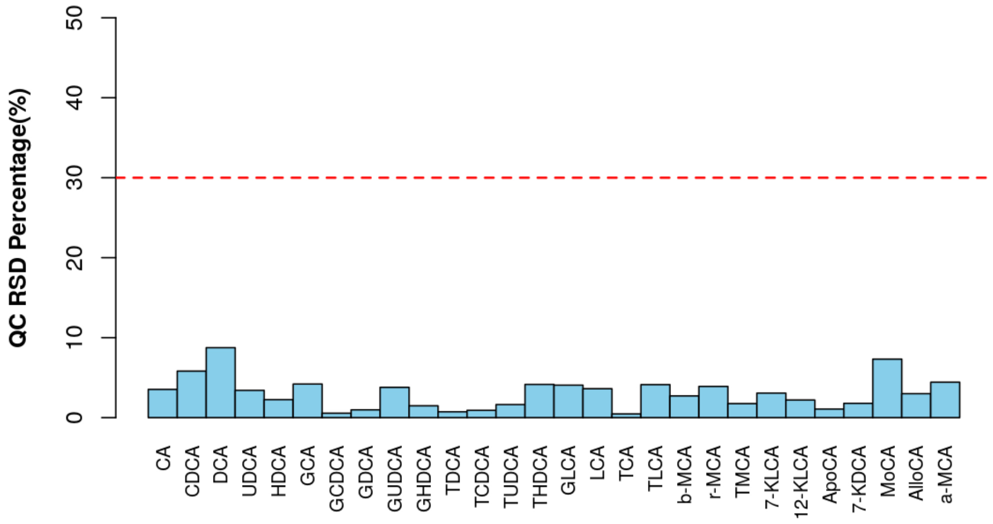

Figure S2. RSD Distribution of Target Substances in QC Samples. An RSD < 30% demonstrates that the analyte data in QC samples is stable and reliable.

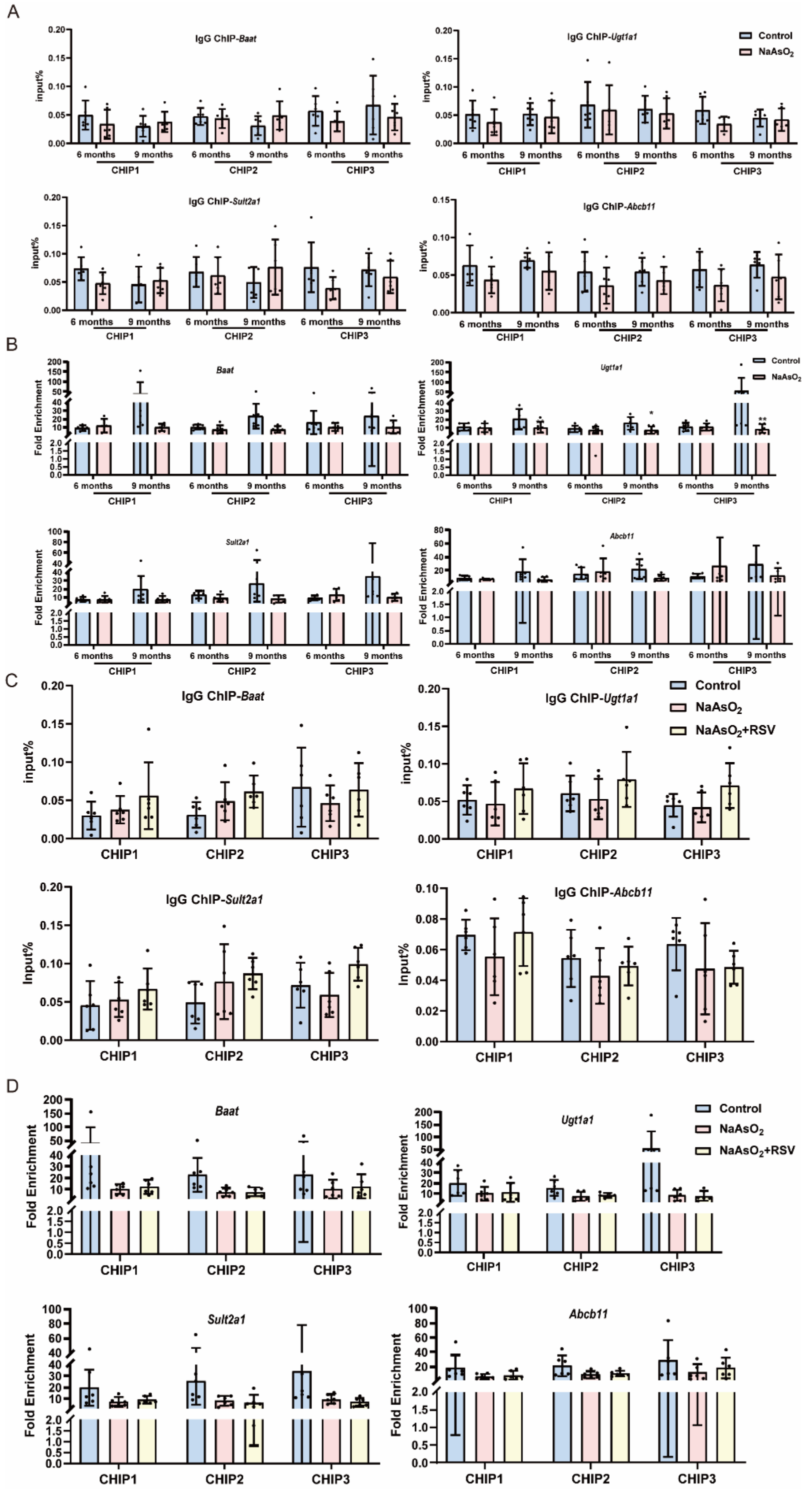

Figure S3. IgG control and fold enrichment in ChIP-qPCR. (A) IgG control in arsenic-induced liver fibrosis rat model. (B) Fold enrichment in arsenic-induced liver fibrosis rat model. (C) IgG control in resveratrol intervention model. (D) Fold enrichment in resveratrol intervention model. Fold enrichment ⩾ 2 indicates specific binding, and higher values represent stronger binding. All data were shown as mean ± SD (n = 6). Independent-samples t-test was used for all predefined pairwise comparisons.
