# Supplementary material for: Resveratrol Alleviates Arsenic-Induced Liver Fibrosis in Rats by Correcting SIRT1-Mediated Disorder of Hepatic Bile Acid Metabolism
Source: Int J Mol Sci. 2026 Jun 5;27(11):5123. doi: 10.3390/ijms27115123 (PMC13257504; doi:10.3390/ijms27115123)
Supplement: Supplementary file 1 [file ijms-27-05123-s001.zip › Tables S1–S14.pdf]

**Table S1. Hepatic Bile Acid Metabolite Alterations in Arsenic-Exposed Rats at Different Time Points**

| Bile acid metabolites (μg/g) | 6 months | | *t* | *p* | 9 months | | *t* | *p* |
|---|---|---|---|---|---|---|---|---|
| | Control (n = 6) | $NaAsO_2$ (n = 6) | | | Control (n = 6) | $NaAsO_2$ (n = 6) | | |
| CA | 0.181±0.050 | 0.298±0.052 | -3.990 | 0.003 [a] | 0.295±0.038 | 1.314±0.428 | -5.812 | 0.002 [a] |
| LCA | 0.026±0.008 | 0.044±0.012 | -3.050 | 0.012 [a] | 0.025±0.002 | 0.049±0.010 | -5.723 | 0.002 [a] |
| TCA | 59.964±12.639 | 121.520±27.423 | -4.994 | < 0.001 [a] | 78.201±29.795 | 168.595±52.898 | -3.647 | 0.004 [a] |
| TCDCA | 2.473±0.372 | 4.372±1.786 | -2.551 | 0.047 [a] | 6.910±1.178 | 18.504±5.716 | -4.866 | 0.004 [a] |
| TDCA | 0.726±0.174 | 1.667±0.174 | -9.361 | < 0.001 [a] | 0.858±0.359 | 3.131±1.599 | -3.397 | 0.017 [a] |
| THDCA | 2.904±0.209 | 6.792±1.914 | -4.946 | 0.004 [a] | 3.306±0.416 | 6.166±1.420 | -4.733 | 0.003 [a] |
| TLCA | 0.264±0.093 | 0.734±0.255 | -4.245 | 0.005 [a] | 0.293±0.138 | 1.315±0.286 | -7.862 | < 0.001 [a] |
| TMCA | 5.379±1.529 | 9.053±2.539 | -3.036 | 0.013 [a] | 5.061±1.576 | 8.545±1.792 | -3.576 | 0.005 [a] |
| CDCA | 0.095±0.006 | 0.117±0.013 | -3.725 | 0.004 [a] | 0.133±0.016 | 0.159±0.032 | -1.781 | 0.105 [a] |
| GCDCA | 0.044±0.018 | 0.315±0.153 | -4.308 | 0.002 [a] | 2.056±1.014 | 1.510±0.965 | 0.955 | 0.362 [a] |
| TUDCA | 0.525±0.349 | 1.327±0.510 | -3.176 | 0.010 [a] | 0.301±0.102 | 0.771±0.651 | -1.748 | 0.138 [a] |
| GCA | 0.961±0.336 | 2.298±1.549 | -2.066 | 0.089 [a] | 23.276±2.734 | 11.419±3.571 | 6.458 | < 0.001 [a] |
| DCA | 0.059±0.004 | 0.066±0.010 | -1.838 | 0.096 [a] | 0.061±0.004 | 0.090±0.033 | -2.162 | 0.081 [a] |
| UDCA | 0.032±0.005 | 0.038±0.015 | -0.973 | 0.353 [a] | 0.037±0.009 | 0.035±0.008 | 0.496 | 0.631 [a] |
| HDCA | 0.367±0.132 | 0.299±0.057 | 1.157 | 0.286 [a] | 0.240 (0.211~0.328) | 0.310 (0.284~0.599) | -2.084 | 0.064 [b] |
| GDCA | 0.056±0.019 | 0.084±0.047 | -1.366 | 0.217 [a] | 0.479±0.093 | 0.414±0.087 | 1.267 | 0.234 [a] |
| GUDCA | 0.010±0.005 | 0.025±0.024 | -1.353 | 0.230 [a] | 0.094±0.016 | 0.156±0.090 | -1.676 | 0.151 [a] |
| GHDCA | 0.160 (0.138~0.301) | 0.191 (0.119~0.353) | -0.246 | 0.811 [b] | 1.091±0.235 | 0.708±0.392 | 2.053 | 0.067 [a] |
| 12-KLCA | 0.056 (0.038~0.266) | 0.140 (0.079~0.168) | -0.720 | 0.496 [b] | 0.147±0.114 | 0.130±0.094 | 0.273 | 0.790 [a] |
| MoCA | 0.057 (0.031~0.175) | 0.051 (0.030~0.081) | 0.683 | 0.510 [b] | 0.028 (0.016~0.105) | 0.040 (0.025~0.071) | -0.247 | 0.810 [b] |
| ApoCA | 0.277 (0.039~3.984) | 0.066 (0.037~0.568) | 0.545 | 0.598 [b] | 1.245±1.333 | 0.050±0.036 | 2.195 | 0.080 [a] |
| AlloCA | 0.177 (0.170~0.223) | 0.167 (0.157~0.199) | 1.086 | 0.303 [b] | 0.186 (0.173~0.201) | 0.226 (0.183~0.386) | -1.525 | 0.158 [b] |
| 7-KDCA | 0.024 (0.021~0.089) | 0.060 (0.046~0.084) | -1.196 | 0.259 [b] | 0.031 (0.020~0.217) | 0.105 (0.034~0.207) | -0.904 | 0.387 [b] |
| 7-KLCA | 0.023 (0.004~0.160) | 0.044 (0.014~0.198) | -0.760 | 0.465 [b] | 0.059 (0.014~0.150) | 0.033 (0.016~0.102) | 0.329 | 0.749 [b] |

[a] Independent samples t-test, the statistical value is *t* value

[b] Log10-transformed data were subjected to an independent samples t-test, the statistical value is *t* value

**Table S2. Resveratrol modulates hepatic bile acid metabolites in arsenic-induced hepatic fibrosis rats**

| Bile acid metabolites (μg/g) | Control (n = 6) | $NaAsO_2$ (n = 6) | $NaAsO_2$ + RSV (n = 6) | *F* | *p* |
|---|---|---|---|---|---|
| CA | 0.295±0.038 | 1.314±0.428 * | 0.858±0.109 # | 23.919 | < 0.001 a |
| LCA | 0.025±0.002 | 0.049±0.010 * | 0.033±0.008 # | 16.579 | < 0.001 a |
| TCA | 78.201±29.795 | 168.595±52.898 * | 87.809±29.704 # | 9.712 | 0.002 a |
| TCDCA | 6.910±1.178 | 18.504±5.716 * | 9.222±2.586 ## | 16.633 | < 0.001 a |
| TDCA | 0.858±0.359 | 3.131±1.599 * | 1.036±0.287 # | 10.385 | 0.001 a |
| THDCA | 3.306±0.416 | 6.166±1.420 * | 2.389±0.886 ## | 23.475 | < 0.001 a |
| TLCA | 0.293±0.138 | 1.315±0.286 ** | 0.291±0.115 ## | 54.820 | < 0.001 a |
| TMCA | 5.061±1.576 | 8.545±1.792 * | 12.348±1.265 ## | 32.785 | < 0.001 a |
| CDCA | 0.133±0.016 | 0.159±0.032 | 0.369±0.046 ## | 88.658 | < 0.001 a |
| GCDCA | 2.056±1.014 | 1.510±0.965 | 4.522±1.770 # | 9.107 | 0.003 a |
| TUDCA | 0.301±0.102 | 0.771±0.651 | 2.901±1.312 ## | 16.016 | < 0.001 a |
| GCA | 23.276±2.734 | 11.419±3.571 ** | 24.252±7.415 ## | 12.216 | < 0.001 a |
| DCA | 0.061±0.004 | 0.090±0.033 | 0.068±0.013 | 3.325 | 0.064 a |
| UDCA | 0.037±0.009 | 0.035±0.008 | 0.176±0.091 ## | 13.796 | 0.631 a |
| HDCA | 0.240 (0.211~0.328) | 0.310 (0.284~0.599) | 0.427 (0.326~0.496) | 3.709 | 0.049 b |
| GDCA | 0.479±0.093 | 0.414±0.087 | 0.631±0.274 | 2.447 | < 0.001 a |
| GUDCA | 0.094±0.016 | 0.156±0.090 | 0.999±0.637 # | 11.124 | 0.001 a |
| GHDCA | 1.091±0.235 | 0.708±0.392 | 2.617±1.211 ## | 10.968 | 0.001 a |
| 12-KLCA | 0.147±0.114 | 0.130±0.094 | 0.202±0.124 | 0.678 | 0.523 a |
| MoCA | 0.028 (0.016~0.105) | 0.040 (0.025~0.071) | 0.061 (0.031~0.138) | 0.798 | 0.469 b |
| ApoCA | 1.245±1.333 | 0.050±0.036 | 0.114±0.083 | 4.557 | 0.028 a |
| AlloCA | 0.186 (0.173~0.201) | 0.226 (0.183~0.386) | 0.395 (0.210~0.480) | 3.470 | 0.058 b |
| 7-KDCA | 0.031 (0.020~0.217) | 0.105 (0.034~0.207) | 0.707 (0.382~1.157) # | 9.540 | 0.002 b |
| 7-KLCA | 0.059 (0.014~0.150) | 0.033 (0.016~0.102) | 0.343 (0.026~0.435) | 2.333 | 0.131 b |

[a] One-way ANOVA, the statistical value is *F* value

[b] Log10-transformed data were subjected to a one-way ANOVA, the statistical value is *F* value

The pairwise p-values were determined by independent-samples t-test. $^{*}p < 0.05$, $^{**}p < 0.001$ vs. Control; $^{\#}p < 0.05$, $^{\#\#}p < 0.001$ vs. $NaAsO_2$

**Table S3. Transcription factors of differential bile acid metabolism-related genes predicted by PROMO database**

| Gene | Transcription factor [Number] |
|---|---|
| *Baat* | AR [T00042]; AT-BP2 [T01016]; C/EBP [T01386]; C/EBPalpha [T00105]; C/EBPalpha [T00107]; C/EBPalpha [T00108]; C/EBPbeta [T00459]; C/EBPbeta [T00581]; C/EBPdelta [T00109]; c-Fos [T00122]; c-Fos [T00123]; c-Fos [T00124]; c-Jun [T00131]; c-Jun [T00132]; c-Jun [T00133]; CREMtau [T01309]; DBP [T00183]; deltaCREB [T01311]; Elk-1 [T00250]; GR [T00333]; HNF-3 [T02277]; HNF-3beta [T01049]; HTF [T05026]; JunD [T00437]; MafG [T01437]; NF-1 [T01298]; PR B [T00697]; STAT4 [T01577]; STAT5A [T04683]; TCF-4E [T02878]; VDR [T00885]; YY1 [T00278]; YY1 [T00915]; ZF5 [T02349] |
| *Ugt1a1* | C/EBPalpha [T00105]; C/EBPalpha [T00107]; C/EBPdelta [T00109]; c-Fos [T00122]; c-Fos [T00123]; c-Fos [T00124]; c-Jun [T00131]; c-Jun [T00132]; c-Jun [T00133]; CREMtau [T01309]; Cutl1 [T02042]; Elk-1 [T00250]; GATA-2 [T01302]; JunD [T00437]; LF-A1 [T00467]; MafG [T01437]; MED8 [T03491]; NF-1 (-like proteins) [T00601]; NF-1 [T00537]; NF-1 [T00539]; STAT5A [T04683]; TGGCA-binding protein [T00832]; TGIF [T04076]; USF-1 [T00875]; VDR [T00885]; YY1 [T00278]; YY1 [T00915]; ZF5 [T02349] |
| *Sult2a1* | AT-BP2 [T01016]; C/EBPalpha [T00105]; C/EBPalpha [T00107]; C/EBPdelta [T00109]; CREMtau [T01309]; DBP [T00183]; Elk-1 [T00250]; HNF-3 [T02277]; HNF-3beta [T02344]; HNF-3beta [T02513]; MafG [T01437]; MATalpha2 [T00487]; NF-AT1 [T01944]; NF-AT3 [T02462]; PPAR-alpha [T00694]; PR B [T00696]; STAT5A [T04683]; TGGCA-binding protein [T00832]; USF2 [T02115]; VDR [T00885]; YY1 [T00865] |
| *Abcb11* | Ttk 69K [T00843]; Pax-2a [T00678]; STAT5A [T04683]; Zic3 [T04671]; MYBAS1 [T05553]; MF3 [T00507]; LIM1 [T04817]; C/EBPalpha [T00107]; MYB2 [T02536]; C/EBP [T01386]; C/EBPdelta [T00109]; PPAR-alpha: RXR-alpha [T05221]; POU1F1a [T00691]; FACB [T02841]; Alfin1 [T04733]; NF-1 [T00537]; NF-1 [T00539]; Pax-6 [T00682]; MyoD [T00526]; JunB [T00436]; HOXA3 [T00378]; AP-2alphaA [T00035]; GA-BF [T00297]; unc-86 [T01882]; TMF [T00835]; TFIID [T00820]; BR-C Z2 [T01478]; Cdx-1 [T01484]; AGL3 [T03025]; MATalpha2 [T00487]; Hb [T00395]; SPF1 [T03975]; Antp [T00026]; Elk-1 [T00250]; ZF5 [T02349]; RC2 [T00724]; VDR [T00885]; Zic1 [T04669]; ABF1 [T00056]; USF2 [T02115]; MafG [T01437]; MNB1a [T01059]; USF-1 [T00875]; HMG I(Y) [T02368]; STAT4 [T01577]; LVc [T00478]; DBP [T00183]; E2 [T00205]; GR [T00333]; muEBP-C2 [T00215]; LCR-F1 [T01599]; TFIIB [T00818]; CREMtau [T01309]; CREMtau1 [T02108]; CREMtau2 [T02109]; MyoD [T01128]; POU3F2 [T00630]; Pax-4a [T02983]; Cart-1 [T03978]; NF-1 [T00538]; R2 [T00712]; Cutl1 [T02042]; C/EBPalpha [T00108]; C/EBPbeta [T00459]; HNF-3 [T02277]; HNF-3beta [T02344]; HNF-3alpha [T00371]; NF-1 [T01298] |

**Table S4. Scoring of protein-protein interaction affinity**

| Node1 | Node2 | Score |
|---|---|---|
| *Sirt1* | *Cebpa* (*C/EBPα*) | 0.738 |
| *Cebpa* (*C/EBPα*) | *Stat5a* | 0.430 |

A score > 0.7 indicates a high-confidence and strong protein-protein interaction.

**Table S5. Internal standard information for bile acid metabolomics**

| Internal Standard Name | Catalog Number | Manufacturer | Target bile acids for quantification |
|---|---|---|---|
| Cholic acid-d4 | C432603 | Toronto Research Chemicals, Canada | CA, MoCA, ApoCA, AlloCA |
| Chenodeoxycholic acid-d4 | C291902 | Toronto Research Chemicals, Canada | CDCA, HDCA |
| Deoxycholic acid-d5 | D232647 | Toronto Research Chemicals, Canada | DCA, 7-KDCA |
| Glycocholic acid-d5 | G641352 | Toronto Research Chemicals, Canada | GCA |
| Glycochenodeoxycholic acid-d7 | G641257 | Toronto Research Chemicals, Canada | GCDCA, GUDCA, GHDCA |
| Ursodeoxycholic acid-d4 | U850007 | Toronto Research Chemicals, Canada | UDCA |
| Lithocholic acid-d4 | L469182 | Toronto Research Chemicals, Canada | LCA, 12-KLCA, 7-KLCA |
| Taurocholic acid-d5 | T008852 | Toronto Research Chemicals, Canada | TCA |
| Taurochenodeoxycholic acid-d5 | T008133 | Toronto Research Chemicals, Canada | TCDCA, THDCA, TMCA, TUDCA |
| Taurolithocholic acid-d5 | T009102 | Toronto Research Chemicals, Canada | TLCA |
| Glycodeoxycholic acid-d4 | 13226 | Isosciences LLC, USA | GDCA |
| Taurodeoxycholic acid-d4 | 13225 | Isosciences LLC, USA | TDCA |

**Table S6. Linear calibration curves, Linear range of detected bile acids**

| Component Name | Mass Info | Retention Time | Linear | R | Linear range （ng/ml） |
|---|---|---|---|---|---|
| CA | 407.4/407.4 | 8.719711952 | y = 0.04414 x + -1.57855 | 0.99935 | 0.5-2500 |
| LCA | 375.3/375.3 | 14.59206548 | y = 0.08402 x + -0.45435 | 0.99914 | 0.5-2500 |
| TCA | 514.4/79.9 | 4.062228721 | y = 0.02734 x + -0.98676 | 0.99834 | 0.5-2500 |
| TCDCA | 498.4/80.0 | 6.172538098 | y = 0.04107 x + -0.87901 | 0.99996 | 0.5-2500 |
| TDCA | 498.4/80.0 | 6.79393994 | y = 0.26419 x + -12.09285 | 0.99945 | 0.5-2500 |
| THDCA | 498.4/79.9 | 3.215778097 | y = 0.13502 x + -2.17184 | 0.99947 | 0.5-2500 |
| TLCA | 482.4/80.0 | 9.11210605 | y = 0.07187 x + -0.28061 | 0.99902 | 0.5-2500 |
| TMCA | 514.4/79.8 | 1.990845141 | y = 0.12588 x + -4.01581 | 0.99912 | 0.5-2500 |
| CDCA | 391.4/391.4 | 11.76776732 | y = 0.09069 x + -2.42350 | 0.99951 | 0.5-2500 |
| GCDCA | 448.4/74.0 | 8.621497932 | y = 0.52105 x + -17.47659 | 0.99953 | 0.5-2500 |
| TUDCA | 498.4/79.8 | 2.89631973 | y = 0.04972 x + -0.66793 | 0.99936 | 0.5-2500 |
| GCA | 464.4/74.0 | 5.99183648 | y = 0.05583 x + 0.00450 | 0.99999 | 0.5-2500 |
| DCA | 391.4/391.4 | 12.13567701 | y = 0.17468 x + -2.25615 | 0.99814 | 0.5-2500 |
| UDCA | 391.4/391.4 | 7.088141858 | y = 0.06585 x + 0.36936 | 0.9996 | 0.5-2500 |
| HDCA | 391.4/391.4 | 8.182297199 | y = 0.04959 x + -2.34983 | 0.99965 | 0.5-2500 |
| GDCA | 448.4/73.9 | 9.382520322 | y = 0.09586 x + -0.23390 | 0.99987 | 0.5-2500 |
| GUDCA | 448.4/73.9 | 4.267892509 | y = 0.04114 x + -0.00205 | 0.99978 | 0.5-2500 |
| GHDCA | 448.4/74.1 | 4.84442342 | y = 0.04222 x + 0.00517 | 0.9997 | 0.5-2500 |
| 12-KLCA | 389.4/389.4 | 8.284905872 | y = 0.00683 x + -0.06994 | 0.9992 | 0.5-2500 |
| MoCA | 391.4/391.4 | 6.023945592 | y = 0.03746 x + 0.57326 | 0.99799 | 0.5-2500 |
| ApoCA | 389.4/389.4 | 10.36667375 | y = 0.03978 x + -4.68549e-4 | 0.9997 | 0.5-2500 |
| AlloCA | 407.4/361.2 | 8.903899755 | y = 0.00927 x + -0.55296 | 0.99934 | 0.5-2500 |
| 7-KDCA | 405.4/405.4 | 4.849211226 | y = 0.06124 x + 0.56204 | 0.99987 | 0.5-2500 |
| 7-KLCA | 389.4/389.4 | 7.77139113 | y = 0.01307 x + 0.00869 | 0.99993 | 0.5-2500 |

**Table S7. Primers used for the real-time PCR assay**

| Primer | Product length (bp) | | Sequence (5'-3') |
|---|---|---|---|
| *Cyp7a1* | 200 | F | ACGCACCTCGCTATTCTCTG |
| | | R | GGCAGGTCATTCAGTTGCAC |
| *Cyp8b1* | 159 | F | AAGGTGGCTCTCTTCCCCTA |
| | | R | CCAGGGCATGTTGTAGTGGT |
| *Cyp7b1* | 124 | F | CAAGCAAATGTCCAGGCAGATA |
| | | R | ATGCGACTGTGGTTTAGTCCT |
| *Cyp27a1* | 153 | F | GCTCCAGGCGCTGAACAA |
| | | R | TTCCACTGCTCCATGCTGTC |
| *Baat* | 159 | F | AGGGGTTGGCATCCTTTCTG |
| | | R | CAGGGTGTAGGCTGGAAGAC |
| *Ugt1a1* | 176 | F | ATGCTGGGAAGCTGTTAGTGA |
| | | R | TTGGAATGGCACAGGGTACTT |
| *Sult2a1* | 156 | F | TGGTTCCTCAAAGGAAATGTTCTA |
| | | R | CCCCAGGAAGTCACATATCTTCT |
| *Slc27a5* (BACS) | 141 | F | GGAACTCTACGGCTCCACAG |
| | | R | GGCTCTGCCGTCTCTATGTC |
| *Slc10a1* (NTCP) | 128 | F | ACAGACAAGGCGCTTAGCAT |
| | | R | AAGGCAACGATCACCCCTTT |
| *Slco1b2* (OATP1B2) | 105 | F | ATGCTTCGTGGGATAGGGGA |
| | | R | TCGGACACTGTCTAGGTGCAT |
| *Abcc2* (MRP2) | 166 | F | TTTGCCCATTATCCGTGCCT |
| | | R | CAAGGCGGAACAGAAGACGA |
| *Abcb11* (BSEP) | 139 | F | TTGTTGGAAGCAGTGGGTGT |
| | | R | CCGATGTTGGAACGGAGGAA |
| *Gapdh* | 153 | F | GGCTCTCTGCTCCTCCC |
| | | R | CCGTTCACACCGACCTT |

**Table S8. ChIP-qPCR Primers with TSS Position Annotation and Predicted Binding Sequences**

| Primer | Product length (bp) | | Sequence (5'-3') | Distance annotation (TSS = 0, upstream as "-") | Predicted binding sequence |
|---|---|---|---|---|---|
| *Baat* | | | | | |
| CHIP-1 | 136 | F | GCCAGTGTTCCTGACTACAGA | -105 ~ -240 bp | TGTTGAAATTCC |
| | | R | GGAGTAGGGGAATAAGGGAGC | | |
| CHIP-2 | 106 | F | AAAATGGCTGCGGTTATAGCTT | -423 ~ -528 bp | GTGCAAAACC |
| | | R | TATGGGGCCTCCTTGTGGT | | |
| CHIP-3 | 100 | F | AGCCAGTATTGAGAACAGAACG | -555 ~ -654 bp | ATTTGAAATAAC |
| | | R | TGTGACACAGCGTGTGTTATTT | | |
| *Ugt1a1* | | | | | |
| CHIP-1 | 122 | F | GGAGGGCTTCCGTGGATAAA | -574 ~ -695 bp | TGTTTCAACATT |
| | | R | AAAAGGGACTGTAAAGTGCCAAG | | |
| CHIP-2 | 105 | F | CTCCTTTCTTTCCAAAATACCGAGT | -614~-718 bp | TGGTAAAATCTT |
| | | R | TCAGAAAGGATTAGCAGAGAGGG | | |
| CHIP-3 | 110 | F | CACTTCGCCATGGAAAACAAAT | -861~-970 bp | TTTGCCAATT |
| | | R | ACTCATGAGAATAAACCTGAGCA | | |
| *Sult2a1* | | | | | |
| CHIP-1 | 100 | F | TCAATGGCTGCTAAGAGATGG | -389 ~ -488 bp | AATTGAAATCCC |
| | | R | AGTTGCTCTTATATGTTGAAGAGTG | | |
| CHIP-2 | 101 | F | TTGTGTGAGTTGAAATTGCTCA | -829 ~ -929 bp | TGTTACAATATT |
| | | R | GCATTCTCTAGTCTGTTGTGAAACT | | |
| CHIP-3 | 145 | F | AATAACCTTTGACTGTGTGTTACAA | -855 ~-999 bp | TTTCACAAC |
| | | R | CCTTGCCCACTGTTCAACTT | | |
| *Abcb11* | | | | | |
| CHIP-1 | 116 | F | TGAGGCTATTGACCAGAGTTCAG | -516 ~ -630 bp | GCAATTGC |
| | | R | CCTCAGTGATCTAGACCCAGC | | |
| CHIP-2 | 120 | F | GATCACTGAGGGCCTGGAAC | -621 ~ -740 bp | TGTCACAAGGGT |
| | | R | AGCCCTTGGTCTTAGAAAATGAC | | |
| CHIP-3 | 100 | F | ACCATGTGGCCCCATTCTAA | -776 ~ -875 bp | TTTCCCAAAGCT |
| | | R | ACTCAAAGCAGGAACCCAAAC | | |

**Table S9. Detailed statistics of outcome measures and differential bile acid metabolites between the 6-month control and 6-month $NaAsO_2$**

| | **6-month control (n = 6)** | **6-month $NaAsO_2$ (n = 6)** | ***P*-value** | ***95% CI*** | **Effect size (*Cohen's d*)** |
|---|---|---|---|---|---|
| Liver-As (μg/g liver) | 0.928±0.574 | 15.578±1.530 | < 0.001 | -16.136 ~ -13.163 | -12.676 |
| ALT (U/g liver) | 330.419±64.055 | 441.026±76.488 | 0.022 | -201.358 ~ -19.856 | -1.568 |
| AST (U/g liver) | 242.100±50.646 | 335.083±31.113 | 0.003 | -147.051 ~ -38.915 | -2.212 |
| α-SMA | 0.117±0.015 | 0.128±0.022 | 0.325 | -0.036 ~ -0.013 | -0.598 |
| COL-IV (μg/g liver) | 58.219±5.554 | 63.953±44.767 | 0.084 | -12.392 ~ -0.924 | -1.108 |
| LN (μg/g liver) | 178.684±9.860 | 193.706±25.503 | 0.208 | -39.894 ~ 9.850 | -0.777 |
| PIIINP (μg/g liver) | 16.526±2.317 | 19.523±3.104 | 0.087 | -6.520 ~ -0.526 | -1.094 |
| HA (μg/g liver) | 11.749±1.047 | 13.314±1.367 | 0.050 | -3.132 ~ 0.001 | -1.285 |
| Masson positive areas (%) | 1.487±0.267 | 1.755±0.444 | 0.234 | -0.739 ~ 0.203 | -0.731 |
| Col I: Col III | 1.217±0.363 | 1.753±0.481 | 0.055 | -1.084 ~ 0.013 | -1.256 |
| TBA (μmol/g liver) | 1.524±0.110 | 1.827±0.168 | 0.004 | -0.486 ~ -0.121 | -2.135 |
| IL-6 (ng/g liver) | 0.173±0.026 | 0.605±0.054 | < 0.001 | -0.486 ~ -0.378 | -10.285 |
| IL-1β (ng/g liver) | 0.102±0.018 | 0.699±0.138 | < 0.001 | -0.741 ~ -0.452 | -6.075 |
| TNF-α (ng/g liver) | 2.229±0.307 | 3.149±0.154 | < 0.001 | -1.232 ~ -0.608 | -3.791 |
| CA (μg/g) | 0.181±0.050 | 0.298±0.052 | 0.003 | -0.182 ~ -0.052 | -2.304 |
| LCA (μg/g) | 0.026±0.008 | 0.044±0.012 | 0.012 | -0.031 ~ -0.005 | -1.761 |
| TCA (μg/g) | 59.964±12.639 | 121.520±27.423 | < 0.001 | -89.022 ~ -34.089 | -2.883 |
| TCDCA (μg/g) | 2.473±0.372 | 4.372±1.786 | 0.047 | -3.769 ~ --0.030 | -1.473 |
| TDCA (μg/g) | 0.726±0.174 | 1.667±0.174 | < 0.001 | -1.165 ~ --0.717 | -5.405 |
| THDCA (μg/g) | 2.904±0.209 | 6.792±1.914 | 0.004 | -5.894 ~ -1.881 | -2.855 |
| TLCA (μg/g) | 0.264±0.093 | 0.734±0.255 | 0.005 | -0.738 ~ - 0.202 | -2.451 |
| TMCA (μg/g) | 5.379±1.529 | 9.053±2.539 | 0.013 | -6.370 ~ -0.977 | -1.753 |

**Table S10. Detailed statistics of molecular indicators between the 6-month control and 6-month $NaAsO_2$**

| | 6-month control (n = 6) | 6-month $NaAsO_2$ (n = 6) | *P*-value | *95% CI* | Effect size (*Cohen's d*) |
|---|---|---|---|---|---|
| **mRNA** | | | | | |
| *Baat* | 0.964±0.268 | 0.280±0.121 | < 0.001 | 0.417 ~ 0.951 | 3.292 |
| *Ugt1a1* | 0.998±0.023 | 0.702±0.221 | 0.022 | 0.064 ~ 0.527 | 1.879 |
| *Sult2a1* | 1.011±0.276 | 0.398±0.209 | 0.001 | 0.298 ~ 0.928 | 2.502 |
| *Abcb11* (BSEP) | 0.971±0.246 | 0.319±0.071 | < 0.001 | 0.419 ~ 0.886 | 3.600 |
| **Protein** | | | | | |
| BAAT | 1.000±0.168 | 0.361±0.074 | < 0.001 | 0.472 ~ 0.806 | 4.922 |
| UGT1A1 | 1.000±0.073 | 0.508±0.168 | < 0.001 | 0.325 ~ 0.658 | 3.798 |
| SULT2A1 | 1.000±0.168 | 0.415±0.205 | < 0.001 | 0.343 ~ 0.826 | 3.112 |
| BSEP | 1.000±0.306 | 0.553±0.302 | 0.029 | 0.056 ~ 0.838 | 1.471 |
| SIRT1 | 1.000±0.154 | 0.612±0.232 | 0.007 | 0.135 ~ 0.642 | 1.974 |
| Ac-C/EBPα | 1.000±0.223 | 4.429±0.890 | < 0.001 | -4.263 ~ -2.594 | -5.284 |
| C/EBPα | 1.000±0.179 | 0.991±0.344 | 0.956 | -0.344 ~ 0.362 | 0.033 |
| **C/EBPα ChIP-*Baat*** | | | | | |
| CHIP1 | 0.407±0.071 | 0.257±0.028 | < 0.001 | 0.079 ~ 0.219 | 2.738 |
| CHIP2 | 0.484±0.095 | 0.288±0.083 | 0.003 | 0.081 ~ 0.311 | 2.198 |
| CHIP3 | 0.674±0.105 | 0.360±0.101 | < 0.001 | 0.181 ~ 0.447 | 3.042 |
| **C/EBPα ChIP-*Ugt1a1*** | | | | | |
| CHIP1 | 0.493±0.102 | 0.288±0.038 | 0.003 | 0.099 ~ 0.314 | 2.675 |
| CHIP2 | 0.530±0.143 | 0.304±0.075 | 0.006 | 0.079 ~ 0.372 | 1.977 |
| CHIP3 | 0.574±0.108 | 0.328±0.037 | 0.002 | 0.133 ~ 0.359 | 3.049 |
| **C/EBPα ChIP-*Sult2a1*** | | | | | |
| CHIP1 | 0.551±0.153 | 0.317±0.041 | 0.012 | 0.073 ~ 0.394 | 2.082 |
| CHIP2 | 0.821±0.212 | 0.471±0.127 | 0.006 | 0.126 ~ 0.576 | 2.007 |
| CHIP3 | 0.628±0.160 | 0.396±0.080 | 0.015 | 0.068 ~ 0.395 | 1.825 |
| **C/EBPα ChIP-*Abcb11* (BSEP)** | | | | | |
| CHIP1 | 0.509±0.100 | 0.293±0.082 | 0.002 | 0.098 ~ 0.333 | 2.353 |
| CHIP2 | 0.666±0.179 | 0.367±0.073 | 0.008 | 0.110 ~ 0.487 | 2.188 |
| CHIP3 | 0.599±0.107 | 0.350±0.066 | < 0.001 | 0.135 ~ 0.362 | 2.806 |

**Table S11. Detailed statistics of outcome measures and differential bile acid metabolites between the 9-month control and 9-month $NaAsO_2$**

| | 9-month control (n = 6) | 9-month $NaAsO_2$ (n = 6) | *P*-value | *95% CI* | Effect size (*Cohen's d*) |
|---|---|---|---|---|---|
| Liver-As (μg/g liver) | 2.848±2.196 | 19.181±1.943 | < 0.001 | -19.000 ~ -13.666 | -7.878 |
| ALT (U/g liver) | 305.220±24.607 | 760.753±67.337 | < 0.001 | -520.747 ~ -390.320 | -8.986 |
| AST (U/g liver) | 220.198±38.129 | 494.625±51.336 | < 0.001 | -332.595 ~ -216.258 | -6.069 |
| α-SMA | 0.114±0.021 | 0.331±0.011 | < 0.001 | -0.239 ~ -0.195 | -12.751 |
| COL-IV (μg/g liver) | 44.552±11.520 | 143.985±5.489 | < 0.001 | -111.696 ~ -87.170 | -11.020 |
| LN (μg/g liver) | 168.792±19.934 | 244.948±10.466 | < 0.001 | -96.636 ~ -55.675 | -4.784 |
| PIIINP (μg/g liver) | 15.825±4.073 | 87.424±14.608 | < 0.001 | -86.893 ~ -56.304 | -6.677 |
| HA (μg/g liver) | 16.768±4.061 | 25.117±2.253 | 0.001 | -12.574 ~ -4.124 | -2.542 |
| Masson positive areas (%) | 1.666±0.455 | 13.584±1.658 | < 0.001 | -13.653 ~ -10.181 | -9.801 |
| Col I: Col III | 1.246±0.431 | 7.528±2.372 | 0.001 | -8.766 ~ -3.798 | -3.685 |
| TBA (μmol/g liver) | 1.534±0.121 | 2.740±0.249 | < 0.001 | -1.472 ~ -0.940 | -6.154 |
| IL-6 (ng/g liver) | 0.162±0.067 | 0.621±0.047 | < 0.001 | -0.534 ~ -0.385 | -7.963 |
| IL-1β (ng/g liver) | 0.124±0.030 | 0.772±0.028 | < 0.001 | -0.686 ~ -0.612 | -22.648 |
| TNF-α (ng/g liver) | 2.191±0.225 | 3.446±0.358 | < 0.001 | -1.640 ~ -0.871 | -4.202 |
| CA (μg/g) | 0.295±0.038 | 1.314±0.428 | 0.002 | -1.468 ~ -0.571 | -3.357 |
| LCA (μg/g) | 0.025±0.002 | 0.049±0.010 | 0.002 | -0.035 ~ -0.014 | -3.304 |
| TCA (μg/g) | 78.201±29.795 | 168.595±52.898 | 0.004 | -145.619 ~ -35.169 | -2.106 |
| TCDCA (μg/g) | 6.910±1.178 | 18.504±5.716 | 0.004 | -17.577 ~ -5.611 | -2.810 |
| TDCA (μg/g) | 0.858±0.359 | 3.131±1.599 | 0.017 | -3.947 ~ -0.599 | -1.961 |
| THDCA (μg/g) | 3.306±0.416 | 6.166±1.420 | 0.003 | -4.347 ~ -1.372 | -2.732 |
| TLCA (μg/g) | 0.293±0.138 | 1.315±0.286 | < 0.001 | -1.311 ~ -0.732 | -4.539 |
| TMCA (μg/g) | 5.061±1.576 | 8.545±1.792 | 0.005 | -5.655 ~ -1.314 | -2.065 |

**Table S12. Detailed statistics of molecular indicators between the 9-month control and 9-month $NaAsO_2$**

| | 9-month control (n = 6) | 9-month $NaAsO_2$ (n = 6) | *P*-value | *95% CI* | Effect size (*Cohen's d*) |
|---|---|---|---|---|---|
| **mRNA** | | | | | |
| *Baat* | 0.978±0.244 | 0.527±0.161 | 0.004 | 0.185 ~ 0.717 | 2.184 |
| *Ugt1a1* | 1.035±0.081 | 0.589±0.277 | 0.010 | 0.159 ~ 0.735 | 2.015 |
| *Sult2a1* | 1.009±0.455 | 0.504±0.141 | 0.041 | 0.029 ~0.982 | 1.501 |
| *Abcb11* (BSEP) | 0.873±0.108 | 0.244±0.093 | < 0.001 | 0.500 ~ 0.758 | 6.251 |
| **Protein** | | | | | |
| BAAT | 1.000±0.158 | 0.420±0.117 | < 0.001 | 0.401 ~ 0.759 | 4.178 |
| UGT1A1 | 1.000±0.362 | 0.515±0.119 | 0.020 | 0.106 ~ 0.864 | 1.801 |
| SULT2A1 | 1.000±0.543 | 0.420±0.212 | 0.048 | 0.008 ~ 1.151 | 1.408 |
| BSEP | 1.000±0.145 | 0.621±0.297 | 0.019 | 0.078 ~ 0.680 | 1.621 |
| SIRT1 | 1.000±0.233 | 0.531±0.290 | 0.007 | 0.131 ~ 0.808 | 1.785 |
| Ac-C/EBPα | 1.000±0.290 | 2.900±0.788 | < 0.001 | -2.664 ~ -1.137 | -3.201 |
| C/EBPα | 1.000±0.300 | 1.173±0.115 | 0.215 | -0.489 ~ -0.142 | -0.764 |
| **C/EBPα ChIP-*Baat*** | | | | | |
| CHIP1 | 0.602±0.126 | 0.347±0.101 | 0.003 | 0.109 ~ 0.402 | 2.238 |
| CHIP2 | 0.539±0.106 | 0.324±0.108 | 0.006 | 0.077 ~ 0.352 | 2.001 |
| CHIP3 | 0.797±0.167 | 0.351±0.099 | < 0.001 | 0.270 ~ 0.623 | 3.256 |
| **C/EBPα ChIP-*Ugt1a1*** | | | | | |
| CHIP1 | 0.552±0.083 | 0.350±0.047 | < 0.001 | 0.116 ~ 0.289 | 3.011 |
| CHIP2 | 0.612±0.034 | 0.339±0.120 | 0.002 | 0.147 ~ 0.399 | 3.090 |
| CHIP3 | 0.696±0.105 | 0.344±0.086 | < 0.001 | 0.229 ~ 0.475 | 3.681 |
| **C/EBPα ChIP-*Sult2a1*** | | | | | |
| CHIP1 | 0.545±0.070 | 0.338±0.089 | 0.001 | 0.104 ~ 0.310 | 2.575 |
| CHIP2 | 0.876±0.152 | 0.463±0.083 | < 0.001 | 0.255 ~ 0.570 | 3.362 |
| CHIP3 | 0.990±0.235 | 0.508±0.130 | 0.001 | 0.237 ~ 0.726 | 2.537 |
| **C/EBPα ChIP-*Abcb11* (BSEP)** | | | | | |
| CHIP1 | 0.645±0.193 | 0.351±0.138 | 0.007 | 0.102 ~ 0.486 | 1.966 |
| CHIP2 | 0.633±0.062 | 0.358±0.138 | 0.001 | 0.137 ~ 0.412 | 2.559 |
| CHIP3 | 0.742±0.124 | 0.372±0.033 | < 0.001 | 0.240 ~ 0.501 | 4.066 |

**Table S13. Detailed statistics of outcome measures and differential bile acid metabolites between 9-month $NaAsO_2$ and $NaAsO_2$ + RSV**

| | 9-month $NaAsO_2$ (n = 6) | $NaAsO_2$ + RSV (n = 6) | *P*-value | *95% CI* | Effect size (Cohen's d) |
|---|---|---|---|---|---|
| Liver-As (μg/g liver) | 19.181±1.943 | 15.076±1.983 | 0.005 | 1.581 ~ 6.631 | 2.092 |
| ALT (U/g liver) | 760.753±67.337 | 409.798±201.379 | 0.002 | 157.805 ~ 544.106 | 2.337 |
| AST (U/g liver) | 494.625±51.336 | 179.239±54.490 | < 0.001 | 247.288 ~ 383.484 | 5.958 |
| α-SMA | 0.331±0.011 | 0.170±0.025 | < 0.001 | 0.136 ~ 0.186 | 8.219 |
| COL-IV (μg/g liver) | 143.985±5.489 | 94.607±16.289 | < 0.001 | 32.289 ~ 666.467 | 4.063 |
| LN (μg/g liver) | 244.948±10.466 | 176.075±43.339 | 0.010 | 23.511 ~ 114.234 | 2.185 |
| PIIINP (μg/g liver) | 87.424±14.608 | 44.789±8.871 | < 0.001 | 27.089 ~ 58.181 | 3.528 |
| HA (μg/g liver) | 25.117±2.253 | 18.475±5.009 | 0.021 | 1.331 ~ 11.953 | 1.710 |
| Masson positive areas (%) | 13.584±1.658 | 4.250±0.856 | < 0.001 | 7.635 ~ 11.030 | 7.072 |
| Col I: Col III | 7.528±2.372 | 3.011±0.967 | 0.002 | 2.186 ~ 6.846 | 2.494 |
| TBA (μmol/g liver) | 2.740±0.249 | 2.036±0.460 | 0.008 | 0.227 ~ 1.179 | 1.901 |
| IL-6 (ng/g liver) | 0.621±0.047 | 0.101±0.040 | < 0.001 | 0.464 ~ 0.576 | 11.944 |
| IL-1β (ng/g liver) | 0.772±0.028 | 0.637±0.091 | 0.013 | 0.040 ~ 0.231 | 2.018 |
| TNF-α (ng/g liver) | 3.446±0.358 | 2.865±0.268 | 0.010 | 0.175 ~ 0.988 | 1.840 |
| CA (μg/g) | 1.314±0.428 | 0.858±0.109 | 0.030 | 0.055 ~ 0.858 | 1.462 |
| LCA (μg/g) | 0.049±0.010 | 0.033±0.008 | 0.013 | 0.004 ~ 0.028 | 1.751 |
| TCA (μg/g) | 168.595±52.898 | 87.809±29.704 | 0.009 | 25.601 ~ 135.971 | 1.883 |
| TCDCA (μg/g) | 18.504±5.716 | 9.222±2.586 | 0.005 | 3.575 ~ 14.988 | 2.092 |
| TDCA (μg/g) | 3.131±1.599 | 1.036±0.287 | 0.023 | 0.420 ~ 3.770 | 1.823 |
| THDCA (μg/g) | 6.166±1.420 | 2.389±0.886 | < 0.001 | 2.254 ~ 5.300 | 3.191 |
| TLCA (μg/g) | 1.315±0.286 | 0.291±0.115 | < 0.001 | 0.743 ~ 1.304 | 4.690 |

**Table S14. Detailed statistics of molecular indicators between the 9-month $NaAsO_2$ and $NaAsO_2$ + RSV**

| | **9-month $NaAsO_2$ (n = 6)** | **$NaAsO_2$ + RSV (n = 6)** | ***P*-value** | ***95% CI*** | **Effect size (Cohen's d)** |
|---|---|---|---|---|---|
| **mRNA** | | | | | |
| *Baat* | 0.527±0.161 | 1.001±0.286 | 0.005 | -0.773 ~ -0.176 | -2.043 |
| *Ugt1a1* | 0.589±0.277 | 1.291±0.426 | 0.007 | -1.163 ~ -0.239 | -1.953 |
| *Sult2a1* | 0.504±0.141 | 1.636±0.639 | 0.007 | -1.801 ~ -0.463 | -2.447 |
| *Abcb11* (BSEP) | 0.244±0.093 | 0.498±0.245 | 0.039 | -0.492 ~ -0.016 | -1.371 |
| **Protein** | | | | | |
| BAAT | 0.572±0.100 | 0.723±0.127 | 0.046 | -0.298 ~ -0.003 | -1.316 |
| UGT1A1 | 0.684±0.261 | 2.287±0.435 | < 0.001 | -2.064 ~ -1.141 | -4.468 |
| SULT2A1 | 0.415±0.109 | 0.952±0.182 | < 0.001 | -0.730 ~ -0.344 | -3.576 |
| BSEP | 0.464±0.092 | 1.088±0.327 | 0.001 | -0.933 ~ -0.314 | -2.593 |
| SIRT1 | 0.515±0.134 | 0.701±0.099 | 0.021 | -0.337 ~ -0.034 | -1.573 |
| Ac-C/EBPα | 4.849±1.284 | 2.000±0.517 | 0.002 | 1.494 ~ 4.202 | 2.909 |
| C/EBPα | 1.253±0.530 | 1.433±0.361 | 0.508 | -0.763 ~ 0.404 | -0.396 |
| **C/EBPα ChIP-*Baat*** | | | | | |
| CHIP1 | 0.347±0.101 | 0.528±0.119 | 0.017 | -0.323 ~ -0.039 | -1.641 |
| CHIP2 | 0.324±0.108 | 0.567±0.161 | 0.012 | -0.419 ~ -0.067 | -1.775 |
| CHIP3 | 0.351±0.099 | 0.546±0.104 | 0.008 | -0.325 ~ -0.064 | -1.918 |
| **C/EBPα ChIP-*Ugt1a1*** | | | | | |
| CHIP1 | 0.350±0.047 | 0.350±0.047 | 0.004 | -0.413 ~ -0.103 | -2.142 |
| CHIP2 | 0.339±0.120 | 0.608±0.164 | 0.009 | -0.519 ~ -0.097 | -1.876 |
| CHIP3 | 0.344±0.086 | 0.715±0.221 | 0.007 | -0.604 ~ -0.138 | -2.211 |
| **C/EBPα ChIP-*Sult2a1*** | | | | | |
| CHIP1 | 0.338±0.089 | 0.561±0.147 | 0.010 | -0.379 ~ -0.067 | -1.835 |
| CHIP2 | 0.463±0.083 | 0.823±0.251 | 0.016 | -0.623 ~ -0.095 | -1.917 |
| CHIP3 | 0.508±0.130 | 0.945±0.239 | 0.003 | -0.685 ~ -0.189 | -2.268 |
| **C/EBPα ChIP-*Abcb11* (BSEP)** | | | | | |
| CHIP1 | 0.351±0.138 | 0.610±0.196 | 0.014 | -0.455 ~ -0.064 | -1.712 |
| CHIP2 | 0.358±0.138 | 0.477±0.038 | 0.091 | -0.264 ~ -0.026 | -1.172 |
| CHIP3 | 0.372±0.033 | 0.731±0.275 | 0.024 | -0.648 ~ -0.071 | -1.833 |
